# Altered resting-state functional connectome in major depressive disorder: a mega-analysis from the PsyMRI consortium

**DOI:** 10.1038/s41398-021-01619-w

**Published:** 2021-10-07

**Authors:** Nooshin Javaheripour, Meng Li, Tara Chand, Axel Krug, Tilo Kircher, Udo Dannlowski, Igor Nenadić, J. Paul Hamilton, Matthew D. Sacchet, Ian H. Gotlib, Henrik Walter, Thomas Frodl, Simone Grimm, Ben J. Harrison, Christian Robert Wolf, Sebastian Olbrich, Guido van Wingen, Lukas Pezawas, Gordon Parker, Matthew P. Hyett, Philipp G. Sämann, Tim Hahn, Olaf Steinsträter, Andreas Jansen, Dilara Yuksel, Robin Kämpe, Christopher G. Davey, Bernhard Meyer, Lucie Bartova, Ilona Croy, Martin Walter, Gerd Wagner

**Affiliations:** 1 Department of Psychiatry and Psychotherapy, Jena University Hospital, Philosophenweg 3, 07743 Jena, Germany; 2 Clinical Affective Neuroimaging Laboratory (CANLAB), Leipziger Str. 44, Building 65, 39120 Magdeburg, Germany; 3 Department of Psychiatry and Psychotherapy, University of Bonn, 53127 Bonn, Germany; 4 Department of Psychiatry and Psychotherapy, Philipps Universität Marburg, Rudolf-Bultmann-Str. 8, 35039 Marburg, Germany; 5 Institute for Translational Psychiatry, University of Münster, 48149 Münster, Germany; 6 Center for Social and Affective Neuroscience, Center for Medical Image Science and Visualization, Department of Biomedical and Clinical Sciences, Linköping University, Linköping, Sweden; 7 Center for Depression, Anxiety, and Stress Research, McLean Hospital, Harvard Medical School, Belmont, MA USA; 8 Department of Psychology, Stanford University, Bldg. 420, Jordan Hall, Stanford, CA 94305 USA; 9 Department of Psychiatry and Psychotherapy CCM, Charité – Universitätsmedizin Berlin, Corporate Member of Freie Universität Berlin, Berlin Institute of Health, Humboldt-Universität zu Berlin, Campus Charité Mitte, Charitéplatz 1, 10117 Berlin, Germany; 10 Department of Psychiatry and Psychotherapy, Otto von Guericke University Magdeburg, Leipzigerstr. 44, 39120 Magdeburg, Germany; 11 Department of Psychiatry and Psychotherapy, CBF, Charité Universitätsmedizin Berlin, 12203 Berlin, Germany; 12 Melbourne Neuropsychiatry Centre, Department of Psychiatry, The University of Melbourne, Victoria, Australia; 13 Center for Psychosocial Medicine, Department of General Psychiatry, University of Heidelberg, Heidelberg, Germany; 14 Department of Psychiatry, Psychotherapy and Psychosomatic, University Zürich, Zürich, Switzerland; 15 Department of Psychiatry, Amsterdam UMC, University of Amsterdam, Amsterdam Neuroscience, Amsterdam, The Netherlands; 16 Department of Psychiatry and Psychotherapy, Medical University of Vienna, Vienna, Austria; 17 School of Psychiatry, AGSM Building, University of New South Wales, Sydney, Australia; 18 School of Psychological Sciences, University of Western Australia, Perth, Australia; 19 Max Planck Institute of Psychiatry, Munich, Germany; 20 Institute for Translational Psychiatry, University of Münster, Münster, Germany; 21 Department of Psychiatry and Psychotherapy & Marburg Center for Mind, Brain and Behavior - MCMBB, Philipps- Universität Marburg, Marburg, Germany; 22 Center for Health Sciences, SRI International, 333 Ravenswood Avenue, Menlo Park, CA USA; 23 Department of Psychiatry, The University of Melbourne, Victoria, Australia; 24 Department of Psychology, Friedrich Schiller University Jena, Jena, Germany; 25 Department of Psychotherapy and Psychosomatic Medicine, TU Dresden, Germany; 26 Leibniz Institute for Neurobiology, Brenneckestr. 6, 39118 Magdeburg, Germany; 27 Department of Psychiatry and Psychotherapy, University Tuebingen, Calwerstraße 14, 72076 Tuebingen, Germany

**Keywords:** Depression, Diagnostic markers

## Abstract

Major depressive disorder (MDD) is associated with abnormal neural circuitry. It can be measured by assessing functional connectivity (FC) at resting-state functional MRI, that may help identifying neural markers of MDD and provide further efficient diagnosis and monitor treatment outcomes. The main aim of the present study is to investigate, in an unbiased way, functional alterations in patients with MDD using a large multi-center dataset from the PsyMRI consortium including 1546 participants from 19 centers (www.psymri.com). After applying strict exclusion criteria, the final sample consisted of 606 MDD patients (age: 35.8 ± 11.9 y.o.; females: 60.7%) and 476 healthy participants (age: 33.3 ± 11.0 y.o.; females: 56.7%). We found significant relative hypoconnectivity within somatosensory motor (SMN), salience (SN) networks and between SMN, SN, dorsal attention (DAN), and visual (VN) networks in MDD patients. No significant differences were detected within the default mode (DMN) and frontoparietal networks (FPN). In addition, alterations in network organization were observed in terms of significantly lower network segregation of SMN in MDD patients. Although medicated patients showed significantly lower FC within DMN, FPN, and SN than unmedicated patients, there were no differences between medicated and unmedicated groups in terms of network organization in SMN. We conclude that the network organization of cortical networks, involved in processing of sensory information, might be a more stable neuroimaging marker for MDD than previously assumed alterations in higher-order neural networks like DMN and FPN.

## Introduction

Major depressive disorder (MDD) is a highly prevalent and disabling neuropsychiatric disease. MDD is associated with lower mood and energy, anhedonia, negative automatic thoughts and ruminations, as well as attention and memory deficits [1]. Furthermore, patients suffering MDD often report sleep disturbances, psychomotor symptoms and somatic symptoms, including general pain [2–6].

Structural and functional magnetic resonance imaging (MRI) have advanced our knowledge about neural alterations linked to MDD. Recent large-scale structural studies mainly found reduced hippocampal volume and thinner cortical gray matter in the orbitofrontal cortex (OFC), anterior (ACC) and posterior cingulate cortex (PCC), and insular cortex (IC) in patients with MDD compared to healthy controls (HC) [7, 8]. These regions are considered as central hubs of the functional brain networks, including the default mode (DMN), frontoparietal (FPN), and salience networks (SN). These networks form a broader triple network governing cognitive and affective operations [9]. Disruption in their functioning are often reported in MDD [10–13].

Functional connectivity analyses of resting-state fMRI (rs-fMRI) data have characterized altered brain networks architecture based on the intrinsic neural activity in the absence of any specified cognitive/affective load in MDD [14, 15]. This approach may identify neural markers to improve MDD diagnosis or tracing predictors for pharmaceutical or psychotherapeutic treatment outcomes [3, 16–18].

Within the triple network, DMN is predominantly characterized by functional coupling of ventromedial prefrontal cortex (vmPFC), PCC and perigenual anterior cingulate cortex (pgACC); and consistently shows “deactivation” during externally directed cognitive tasks compared to baseline [19, 20]. In turn, DMN has been posited to be involved in mind-wandering and processing of self-related information [15]. Of note, DMN regions showed activation mostly during processing of internally directed stimuli, and tasks measuring social interactions [21]. Abnormal functioning of the DMN was frequently related to negative self-referential ruminative thinking as the primary MDD symptoms [16, 22]. Therefore, many studies have focused on the neural alteration of DMN and often reported hyperconnectivity within DMN in depressed patients as an indicator of increased ruminative thinking in MDD [23, 24].

The FPN was shown to exhibit lower FC in patients with MDD than in healthy participants [23, 25]. This network consists of dorsolateral prefrontal cortex (DLPFC), frontal operculum, and superior parietal cortex. Dysregulation of this network may disrupt goal-directed cognition, and working memory [26].

Previous rs-fMRI studies also reported abnormal FC within the above mentioned higher-ordered networks, i.e., between DMN, SN, and FPN, which was related to altered cognitive functioning in patients with MDD [10, 27]. SN comprises anterior insula (aI) and dorsal ACC (dACC) and has been posited to map external salient stimuli and interoceptive signals [28, 29]. In addition, SN integrates these signals mainly through the dACC, and helps to allocate brain resources between the DMN and the FPN [30].

Thus, numerous rs-FMRI studies have found dysfunctional coupling within and between specific higher-order networks in MDD [31]; yet, replication of these findings is often unfeasible due to differences in the study design, sample characteristics, data acquisition protocols, statistical analyses, and thresholding; and importantly most of these studies had small sample sizes that may lead to false positive and ungeneralizable results [32].

Investigating neural markers of MDD using meta-analyses and mega-analyses may help to overcome the sampling and statistical biases. A coordinate-based meta-analysis has found hyperconnectivity within DMN as well as hypoconnectivity within FPN in MDD patients comparing to healthy controls [33]. However, this study was based on the collected statistical maps from the included studies, and thus might have failed to capture specific image information i.e., full spatial characteristics and within subject variability [34]. Furthermore, this meta-analysis included adolescents, middle-aged, and elderly adults. A recent mega-analytic study analyzed raw resting-state fMRI datasets in a more homogenous sample, and showed in contrast to the meta-analysis hypoconnectivity within DMN and no abnormalities within FPN in patients with MDD compared to HC [35]. In a further exploratory analysis, this study observed hyperconnectivity within visual (VN) and somatosensory motor networks (SMN). Differences in analytic methods and sample characteristics may explain the contradictory results of these two currently largest rs-fMRI studies in MDD until now.

Identifying consistent resting-state patterns is crucial to determine stable MDD-specific neural markers. Thus, the ultimate goal of the present study was to identify dysfunctional networks using an unbiased (i.e., data driven) approach in adult patients with MDD using a large sample size of 1546 participants from a collaboration of 19 clinical research centers (http://psymri.org/). To achieve this goal, we investigated altered resting-state FC within and between seven main brain networks (i.e., DMN, SN, FPN, SMN, DAN, VN, and limbic (LN)), as well as on the nodal level. These networks were selected based on the Schaefer parcellation scheme, which is the most homogeneous parcellation to date, and is aligned with cortical boundaries determined by histology and visuotopic fMRI generated from 1489 participants [36]. According to the aforementioned studies, we were expecting to find aberrant FC mostly in DMN and FPN in patients with MDD compared to healthy controls.

Furthermore, the FCs are organized in networks that have denser interconnections in the synchronicity of the balanced global integration. Presumably, the networks function effectively by maintaining the balance of within network connections relative to the whole brain networks interactions [37]. Therefore, as an exploratory analysis, we used segregation measure as a summary statistic to investigate the group differences in the functional organization of networks and to investigate putative age-related effects on it, as was recently demonstrated for structural brain abnormalities in MDD [38]. This graph theoretic measure explicates the ratio of within-network connectivity relative to connectivity between regions of that network to other regions of the brain, validated by several studies [39–41]. It was shown to be strongly associated with many other traditional graph measures and seems to capture age-related effects on the brain network organization more strongly than the other measures [39].

As the final goal, we aimed to explore the association between identified FC abnormalities and different level of depression severity, which might facilitate future studies to monitor the treatment outcomes, and thus having a better response prediction to treatments or relapses [2, 25].

## Materials and methods

Data for the current study were provided by PsyMRI consortium (http://psymri.org/) with contribution of 19 clinical research centers with a total number of 1546 participants (MDD: 841, HC: 698). As an inclusion criterion, patients in each center had to fulfill an MDD diagnosis according to SCID-I or MINI for DSM-IV Axis I Disorders. Exclusion criteria were a lifetime history of schizophrenia, a lifetime history of severe head trauma or central nervous system disorder and a history of alcohol/substance abuse or dependence. This study was approved by the respective local ethics committees. Informed written consent was obtained from all participants prior to their participation.

We applied conservative exclusion criteria to control for site and demographic variation. We only included adults (18–65 years) and excluded subjects with excessive head motion [42] (see the Supplemental material for details and PRISMA flow-diagram [43] in Supplemental Fig. 1). We finally included 1082 participants in the key analysis (MDD: 606, HC: 476) as reported in Table 1, Supplemental Tables 1 and 2 as well as in Supplemental Fig. 2. For preprocessing of the fMRI data, we followed the standard protocol as detailed in supplemental material.

**Table 1:** Demographic information of the included study participants.

	Controls	Patients	Total
	(*N* = 476)	(*N* = 606)	(*N* = 1082)
*Age*			
Mean (SD)	33.3 (11.0)	35.8 (11.9)	34.7 (11.6)
*Gender*			
Female	270 (56.7%)	368 (60.7%)	638 (59.0%)
Male	206 (43.3%)	238 (39.3%)	444 (41.0%)
*Antidepressant medication*			
Yes		383 (63.2%)	
No		220 (36.3%)	
Not reported		3 (0.5%)	
*Depression severity status*			
Remitted		141 (23.3%)	
Mild		102 (16.8%)	
Moderate		201 (33.2%)	
Severe		101 (16.7%)	
Very severe		39 (6.4%)	
Not reported		22 (3.6%)	

We applied conservative exclusion criteria to control for site and demographic variations (see PRISMA flow diagram in Supplemental Fig. 1). Our analyses were restricted to datasets acquired with the 3 Tesla scanners (the scanner information, acquisition details and demographic characteristics for each site can be found in supplemental Tables 1 and 2). The age band was limited to the adults (18–65 years) to reduce the variance in FC due to potential confounding neurodegenerative disorders in the elderlies or comorbid developmental conditions in adolescents. Our exclusion criteria at the subject level were: subject drop-outs reported by study centers, incomplete or missing neuroimaging and demographic data, excessive head motion (mean frame-wise displacement (mean FD > 0.55). Patients and healthy subjects were not significantly different in terms of age and gender as indicated by Kruskal–Wallis and Chi-squared tests. Depression severity across included studies were acquired using different standard questionnaires. The total scores of used questionnaires from different centers were converted to five depression severity categories (see Supplemental Table 3 for detailed demographic information): remitted, mild, moderate, severe, and very severe. The average scores of site-specific questionnaires are reported in Supplemental Table 4.

For further exploratory analysis, patients were categorized based on the level of depression severity. Since it was assessed across included studies using different standard depression scales, the total scores of these questionnaires from different centers were converted to five depression severity categories (supplemental Table 3): remitted, mild, moderate, severe and very severe [44, 45]. The average scores of site-specific questionnaires are reported in Supplemental Table 4.

### Brain parcellation, definition of networks, and network segregation

We utilized a parcellation scheme with 400 cortical parcels to have both the functional homogeneity and cortical areas differentiation. This scheme was used to extract the average time courses for each parcel. The Pearson correlation coefficient matrix among all parcels was calculated and Fisher Z-transformation was applied for each participant [36].

To investigate differences in functional connectivity at the network level, we firstly compared the averaged FCs within the seven cortical brain networks (VN, SMN, DAN, SN, LN, FPN, and DMN) [46] between patients with MDD and HCs. Secondly, we compared FCs at the nodal level from all 400 ROIs (79,800 FCs) between MDD patients and healthy controls.

To calculate the network organization using segregation metric, we averaged all positive z-transformed FC values within a network ($\bar Z$_n_). Then, averaged FC values between nodes of that network to all nodes in other networks ($\bar Z$_a_) was subtracted from ($\bar Z$_n_) and divided by averaged FC values of that network ($\bar Z$_n_) [39], i.e., network segregation = $\frac{\bar Z_{{\rm{n}}}-\bar Z_{{\rm{a}}}}{\bar Z_{{\rm{n}}}}$.

### Statistical models

To compare patients with MDD and healthy controls, we used linear mixed-effect model (LMER) using LME4 lme4_1.1-23 package [47] in R (version 4.0.0). We defined a factor *group* (MDD, HC), and variables age, sex, mean framewise displacement (FD) as fixed effect variables, and *site* as a random variable. The FC metrics, i.e., (i) averaged FCs within each network, (ii) pair-wise FCs, and (iii) network segregation metric were set as dependent variables.

To have comparable normalized variables, the dependent variables were inverse normal transformed prior to their use in the linear mixed models [48]. To correct for multiple comparisons, we adjusted the α-level for the comparisons regarding seven network FCs and segregation measures by the Bonferroni method. Additionally, to compare estimated mean differences of the averaged FC between patients and controls within a network across each PsyMRI center, we applied this model separately for each site.

Comparing single pair-wise FCs (*n* = 79,800), we utilized false discovery rate (FDR) correction to balance between the likelihood of making a type I error and the probability of making a type II error [49]. To calculate the effect sizes of linear mixed models in this study we used EMAtools R package [50]. To check the results of the mega-analysis, we additionally performed a meta-analysis with FCs from single centers. Fixed-effects linear model was used at the site level to estimate group differences adjusted for age, gender, and mean FD covariates to control for within site variability. The estimated mean of group differences and corresponding standard deviations have been used to perform meta-analysis using inverse variance-weighted fixed-effect meta-analysis [51–53].

For the analysis of depression severity, we set the categorical variable *severity* (remitted, mild, moderate, severe, and very severe) as a factor. To investigate the effect of antidepressant medication on FC and segregation metrics, we compared medicated and unmedicated patients. To investigate age-related effects on segregation metric, we calculated the interaction of age and factor group (MDD, HC) with the segregation metric in the linear regression model. The slopes were calculated using emtrends as implemented in emmeans_1.6.0 package in R, and then the differences between slopes of predicted regression lines for each group were compared.

## Results

### Differences in FC at the network level

Comparing averaged FCs between MDD patients and healthy controls, we found *lower* FCs within SN (*t* = 3.1, *p*-value = 0.002, Bonf. corr.) and SMN (*t* = 4.3, *p*-value < 0.001, Bonf. corr.) in MDD patients (Table 2). The mean FC within DMN was also lower in MDD patients relative to healthy controls. However, this result was not statistically significant after Bonferroni correction (*t* = 2.0, *p*-value = 0.043). The forest plot displaying the results of the FC analyses separately for each site, also indicated mostly lower averaged FCs within these three networks in each center (Supplemental Figs. 3, 4, and 5).

**Table 2:** Group differences in averaged FCs within each cortical network.

Network	Adjusted mean: controls	Adjusted mean: patients	*t*-value	Cohen’s *d*	Uncorrected *p*-value
FPN	0.057	−0.014	1.381	0.085	n.s
DMN	0.057	−0.046	2.021	0.124	0.043
DAN	0.073	−0.053	2.61	0.16	0.009
LN	0.041	−0.038	1.59	0.097	n.s
SN	0.082	−0.070	3.073	0.188	0.002^a^
SMN	0.112	−0.098	4.283	0.262	<0.001^a^
VN	0.057	−0.063	2.483	0.152	0.013

The means of averaged FCs for each group (adjusted for age, gender, mean frame-wise displacement, site), *t*-values, Cohen’s *d* and uncorrected *p*-values of FCs are reported for the comparison between patients and healthy subjects at the network level.

*FPN* frontoparietal network, *DMN* default mode network, *DAN* dorsal attention network, *LN* limbic network, *SN* salience network, *SMN* somatosensory motor network, *VN* visual network.

^a^Significant *p*-value after Bonferroni correction for multiple comparisons.

### Differences in FC at the nodal level

**Fig. 1:**
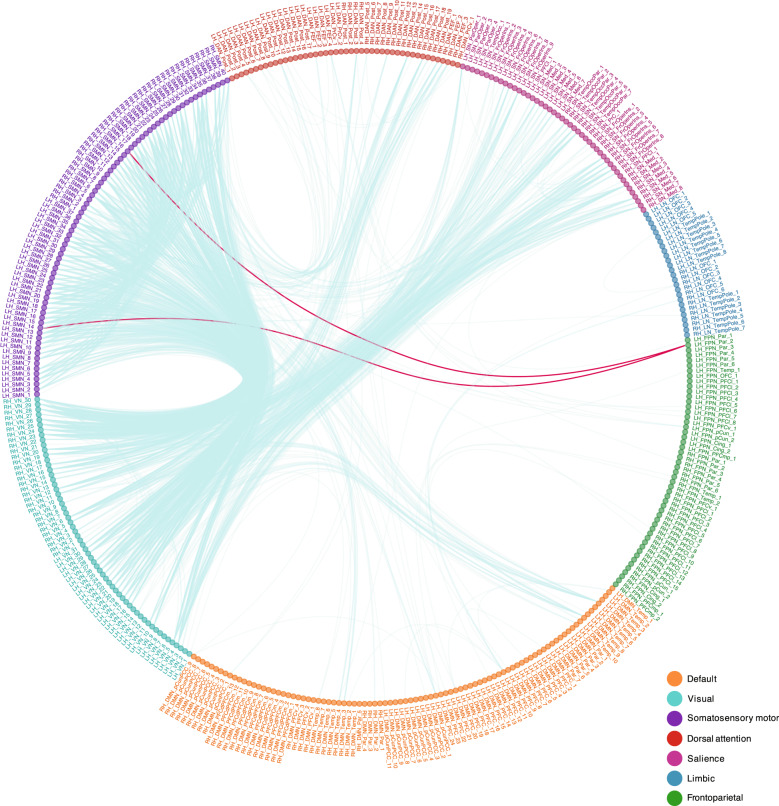
Group differences in FCs between 400 nodes of the seven cortical networks. In this figure each color in the circle represents a network. The blue lines show lower FC in patients with MDD compared to HCs and the red lines show greater FC in patients with MDD compared to HCs. The FC differences between patients with MDD and healthy controls as estimated mean differences regarding 400 nodes (79,800 FC) were obtained from linear mixed effect model (LMER) with age, gender, mean framewise displacement as fixed variable and site as random variable. The false discovery rate (FDR) was used to correct for multiple comparisons (*p*-value < 0.05).

Comparing all 79,800 FCs derived from 400 parcels the majority of significant FCs after FDR correction (1824 significant FCs, corrected *p*-value < 0.05) indicated a relative *hypoconnectivity* within and between several networks in MDD as illustrated in the Fig. 1 (see also Supplemental Fig. 6). The relative *hypoconnectivity* was mostly present within SMN and SN. However, we also detected lower FCs between SMN, VN, SN, and DAN as well as from some few temporal lobe regions in DMN to SMN. Although it is negligible, we found relative *hyperconnectivity* between a temporal region within FPN and two regions within SMN. The meta-analysis over 13 included centers showed similar results as the mega-analysis after FDR correction (*p*-value < 0.05) (Supplemental Fig. 7).

### Network organization

Comparing the seven brain networks between MDD patients and HCs regarding their network segregation, we only found significantly lower segregation of SMN in patients with MDD comparing to HC (*t*-value = 3.707, *p*-value < 0.001, Bonf. corr.) (Table 3).

**Table 3:** Group differences in segregation metrics of each cortical network.

Network	Adjusted mean: controls	Adjusted mean: patients	*t*-value	Cohen’s *d*	Uncorrected *p*-value
FPN	0.039	0.040	−0.023	−0.001	n.s
DMN	0.003	0.009	−0.124	−0.008	n.s
DAN	0.023	0.039	−0.332	−0.02	n.s
LN	−0.076	−0.072	−0.095	−0.006	n.s
SN	0.035	0.021	0.304	0.019	n.s
SMN	0.134	−0.043	3.707	0.227	<0.001^a^
VN	0.002	0.020	−0.381	−0.023	n.s

^a^Significant *p*-value after Bonferroni correction for multiple comparisons.

The means of network segregation for each group (adjusted for age, gender, mean frame-wise displacement, site), *t*-values, Cohen’s *d*, and uncorrected *p*-values of network segregations are reported for the comparison between patients and healthy subjects.

*FPN* frontoparietal network, *DMN* default mode network, *DAN* dorsal attention network, *LN* limbic network, *SN* salience network, *SMN* somatosensory motor network, *VN* visual network.

Furthermore, SMN segregation decreased significantly with age in patients (*t*-value = −5.193, *p*-value = <0.001), but not in healthy controls (*t*-value = −1.509, *p*-value = 0.131). Comparing the slopes of this association between healthy controls and MDD patients, patients showed a significantly greater negative slope than healthy controls (*p*-value = 0.035, Supplemental Fig. 8). Thus, this finding indicates faster SMN segregation decline with aging in MDD patients compared to healthy controls.

### Depression severity

**Fig. 2:**
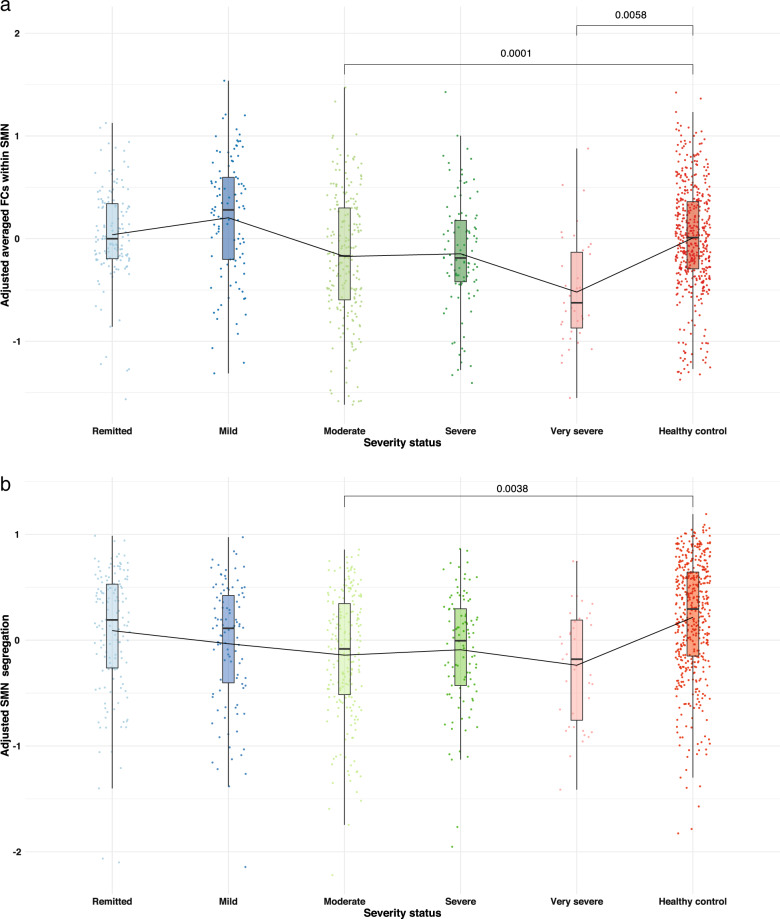
Bar-plots of the averaged FCs for different level of depression severity within SMN and of SMN segregation. The means reported in these bar-plots are adjusted for age, gender, mean framewise displacement and site according to the linear mixed model and the reported *p*-values are significant after Bonferroni correction for multiple comparisons (*p* < 0.05). **a** The averaged FCs within somatosensory network (SMN) for patients’ groups defined according to depression severity and healthy participants. **b** The averaged SMN segregation for patients’ groups defined according to depression severity and healthy participants.

Comparing the five levels of depression severity in MDD patients, we did not find any overall significant differences in network FC and segregation (after Bonferroni correction) in patients with different depression severity. However, comparing separately these patient groups to healthy controls (Fig. 2), significantly *lower averaged FC* was only observed within SMN in moderate and very severely depressed patients (moderate severity: *p*-value <0.001 and very severe: *p*-value = 0.006, Bonf. corr.). Significantly *lower segregation* was only found in SMN in patients with moderate depression severity relative to healthy controls (*p*-value = 0.004, Bonf. corr.).

### Medication status

Comparing medicated and unmedicated patients (see Supplemental Table 5 for demographic characteristics) no differences were found regarding depression severity (*χ*² = 391.8, *p*-value = 1, using Monte-Carlo simulation, replicated for *B* = 10^3^ samples). Comparing both groups regarding network FC and networks segregation, we found that medicated patients exhibited lower FC in DMN, FPN, SN, and SMN (DMN: *p*-value = 0.0028, FPN: 0.0032, SN: *p*-value = 0.0005 and SMN: *p*-value = 0.0007, Bonf. corr.). Higher segregation in DAN was found in medicated compared to unmedicated patients (DAN: *p*-value = 0.0046, Bonf. corr.) (Supplemental Tables 6 and 7). No significant differences were detected regarding SMN segregation between medicated and unmedicated patients.

## Discussion

The main finding of the present mega-analytic study is that effects are small and reduced throughout the examined FCs at the network and nodal level in MDD, only attaining significance in sensory networks beyond those hypothesized. Furthermore, only SMN exhibited significantly lower segregation in patients relative to healthy participants. We also observed that both SMN alterations in FC and network segregation, were more strongly pronounced in patients with moderate and very severe depressive symptoms, possibly indicating a state or severity dependency of these alterations.

Although the difference in DMN-FC was not significant after correcting for multiple comparisons, we found hypoconnectivity in patients within DMN at the network level. This finding is in agreement with the previous mega-analytic study [35]. In accord with this study, we did not detect any significant alterations in FPN in patients with MDD. In pair-wise FCs comparisons between 400 parcels only few significantly altered connections were observed within DMN and FPN or between DMN and FPN relative to other networks. The hypoconnectivity was detected mainly between temporal lobe regions of DMN and SMN as well as VN regions. These findings contradict the results of a previous meta-analysis showing relative hyperconnectivity between DMN regions [33]. This study integrated seed-based studies, and beside diverging analytic strategy, further reasons for the discrepant findings might reflect different characteristics of the included samples, such as age range. We only included here adults between 18–65 years old to exclude potential effects due to age-related neural development in adolescents or neurodegeneration in older adults [54].

### Differences in the default mode and frontoparietal networks

Given the suggested relationship between self-related processing and DMN activity/connectivity, it is conceivable that not all patients show increased level of ruminative thinking during the resting-state fMRI scanning. Previous studies found a correlation between increased FC in DMN and elevated rumination trait [55], indicating that the robust detection of DMN alterations may require an active state of self-related processing.

Abnormal functioning of specific FPN regions in MDD patients was often demonstrated in task-based fMRI studies while patients are engaged in cognitive tasks [16, 56, 57]. Thus, one possible explanation for the negative finding in our study is that the rs-fMRI approach may not be sufficiently sensitive to detect abnormal functioning in this network as long as no cognitive strain is exerted.

An additional analysis of the impact of depression severity on FC did not reveal any significant differences within DMN and FPN connectivity across different severity categories compared to healthy subjects. This finding further points toward the weak association between alterations in these networks and MDD in general.

Previous rs-fMRI studies were often biased by selecting single spherical ROIs, using different spatial maps between groups derived from group ICA in small sample sizes; or only focusing on certain part of a network, i.e., posterior or anterior DMN [23, 31, 58, 59]. Thus, statistical effects of DMN and FPN alteration in MDD might be overestimated.

Another explanation might be that both DMN and FPN are altered only in a subgroup of patients with specific depressive symptoms. As reported by Drysdale et al. different clusters of MDD symptoms seem to be associated with distinct within and between network FC alterations [14].

The alterations in DMN and FPN in depressed patients might be also obscured by the effect of antidepressant medication on FC. Medicated patients showed significantly lower FC in DMN and FPN than unmedicated patients. Likewise, lower FC within DMN was shown in recent studies in medicated compared to the drug-naïve first-episode patients with MDD [35, 60].

Based on the present results, alterations in resting-state FC of the DMN and FPN cannot be considered to be a stable neural marker of MDD in general, as previously assumed, which can be partly based on publication and confirmation bias. Moreover, considering the confounding effects of medication, depression subtypes and severity, the concept of biomarker stability might be not useful in the context of psychiatric diseases.

### Differences in the somatosensory and salience networks

Regarding our main finding of highly significant hypoconnectivity within SMN and SN, previous studies have demonstrated abnormal connectivity within and between regions in SMN and SN in MDD [61–63]. The recent mega-analysis also indicated reduced connectivity in SMN [35]. Besides FC differences, another large-sample rs-fMRI study reported lowered resting-state activity in sensorimotor, but also frontal and visual regions using fractional amplitude low-frequency fluctuation (fALFF) [64].

Reduced connectivity of SMN to the dopaminergic ventral tegmental area (VTA) and posterior insula was associated with disincentive behaviors [11]. Other studies related reduced FCs between SMN, SN, and VN to inability to distract attention from negative stimuli [65]. Furthermore, abnormal FC from SMN to VN, DAN, and DMN has been shown to correlate with both psychomotor retardation and agitation in depressive disorders [66].

Regions within SMN, such as somatosensory cortex, and ACC and insular cortices that constitute SN, play a central role in the body awareness and the complex experience of pain [67, 68]. Patients with MDD were consistently shown to have diminished activation in dACC, somatosensory cortex and posterior insula to pain stimuli [69]. Intriguingly, in a large-scale community study a high rate (50%) of pain symptoms in patients with MDD was documented [70, 71]. Therefore, in some recent publications it was suggested to add pain symptoms to the diagnostic criteria for MDD [72]. Altered FCs within SMN and between SMN and SN might be associated with misinterpreting specific interoceptive sensations leading for example to pain symptoms and pain unpleasantness in depressed patients [6, 73].

Crucially, anterior insula as the main hub of SN converges both external stimuli information from sensory networks and interoceptive signal [29]. The interconnection of this node to another core node of SN (dACC) enables this network to regulate DMN and FPN activities or sensory network responses [28]. Deficits in the connectivity of SN and SMN might lead to abnormalities in SMN. Psychomotor retardation as occurring in severe depression could be the behavioral correlate of this finding [9]. A recent meta-analysis provided evidence of common FC alterations between SN and DMN as well as FPN in several psychiatric disorders, however without being able to show disorder-specific contribution to these abnormalities in FC [13]. Thus, whether the observed alterations in SMN and SN represent a specific MDD feature, have to be shown in futures studies.

### Differences in brain organization

In addition to abnormal functional connectivity of the SMN, we also demonstrated decreased SMN segregation in MDD. This functional brain organization indicates the balance between functional specialization (connections within a network) and integration (connections between networks), which is crucial for neural network functioning and cognitive/affective processing. This is a new finding and shows not only that within-SMN connectivity is disrupted in MDD, but also that the global functional embedding of this network is altered in terms of a diminished compartmentation. Hence, it will be interesting to investigate in future studies the significance of this finding in relation to specific depressive symptoms.

Lower network segregation was found in some studies to be associated with aging. Higher network segregation seems to represent a more normative state in healthy young and middle-aged adults [39, 41]. In the present study, SMN segregation decreased with age only in patients. A significant difference in regression slopes between patients and controls indicated an accelerated age-related decline in the SMN segregation in MDD patients compared to healthy controls, pointing toward progressive changes in brain network organization. As presumed by a previous study, predicted brain age is higher in MDD patients utilizing structural data [38]. Thus, our data on the SMN could reflect another novel marker of accelerated brain age in MDD.

Finally, we only found reduced FC within SMN as well as lower SMN segregation in patients with moderate and very severe depressive symptoms. One explanation for this finding in addition to psychomotor retardation in severe depression might be that the more severe depression, the more likely it is to be associated with other somatic symptoms. Since we only had the total scores of depression rating scales, we were not able to perform such an analysis.

Findings regarding within-SMN FC and SMN segregation were significantly reduced only in severe MDD, but not in patients with mild depressive symptoms highlights a potential of this marker to be state-dependent, which deserves attention in future longitudinal studies. Furthermore, the network organization in SMN were similar in both medicated and unmedicated groups, indicating that this finding was not influenced by antidepressant treatment.

Given the recent large-sample structural studies regarding the significant association between body mass index (BMI) as well as early-life adversity (ELA) and cortical/subcortical regions [74, 75], it is conceivable that the observed structural alterations may also have an impact on functional activation as well as functional connectivity. Thus, these associations should be investigated in future mega-analyses. Unfortunately, this information was not available in the present study.

### Limitations

As a limitation of the present study, we were not able to relate possible symptoms clusters to the observed alterations in FC, and more in-depth analyses are needed to relate FC markers to MDD subgroups. Even though we performed a comparison regarding current psychopharmacological medication, the absence of specific information did not allow us to perform further sub-analysis regarding these potentially confounding factors. No information was available regarding the wash-out period, being drug-naïve, current non-pharmacological therapies like ECT and TMS, having a treatment-resistant depression or recurrent MDD, BMI, disease comorbidity, early-life adversity and education. Our findings may not be generalized to adolescence or geriatric depressive disorder. Finally, we could not extend our findings to subcortical regions, due to the applied parcellation scheme.

## Conclusion

Our main findings underscore FC alterations within sensorimotor and salience networks, as well as to other networks. The central role of the salience network for receiving and processing external and internal signals could be related to typical cognitive and behavioral symptoms of MDD, for example increased attention to interoceptive or negative information or reduced capacity to focus on external tasks. Sensorimotor network abnormalities were more pronounced in more severe depression, pointing toward a potential neural marker for monitoring the disease state. With respect to network organization, patients were comparable in SMN irrespective of medication status. Explicitly, it could be a more prominent neuroimaging marker to differentiate MDD patients from healthy controls, which also might signify acceleration of the brain age in patients diagnosed with MDD compared to healthy controls.

## Supplementary information

Supplemental Material

Supplemental Figure 1

Supplemental Figure 2

Supplemental Figure 3

Supplemental Figure 4

Supplemental Figure 5

Supplemental Figure 6

Supplemental Figure 7

Supplemental Figure 8
