# Supplementary material for: Altered resting-state functional connectome in major depressive disorder: a mega-analysis from the PsyMRI consortium
Source: Transl Psychiatry. 2021 Oct 7;11:511. doi: 10.1038/s41398-021-01619-w (PMC8497531; doi:10.1038/s41398-021-01619-w)
Supplement: Supplementary file 1 — Supplemental Material [file 41398_2021_1619_MOESM1_ESM.docx]

**Supplemental Material**

# Title: **Altered resting-state functional connectome in major depressive disorder: a mega-analysis from the PsyMRI consortium**

Running title: **Functional connectome in major depressive disorder**

1 Nooshin Javaheripour (Ms.) MA Department of Psychiatry and Psychotherapy, Jena University Hospital, Philosophenweg 3, 07743 Jena, Germany & Clinical Affective Neuroimaging Laboratory (CANLAB), Leipziger Str. 44, Building 65, 39120 Magdeburg, Germany +49 36419390463 Nooshin.Javaheripour@med.uni-jena.de

2 Meng Li (Dr) PhD Department of Psychiatry and Psychotherapy, Jena University Hospital, Philosophenweg 3, 07743 Jena, Germany & Clinical Affective Neuroimaging Laboratory (CANLAB), Leipziger Str. 44, Building 65, 39120 Magdeburg, Germany +49 3641 9390485 meng@canlab.de

3 Tara Chand (Mr.). MS Department of Psychiatry and Psychotherapy, Jena University Hospital, Philosophenweg 3, 07743 Jena, Germany & Clinical Affective Neuroimaging Laboratory (CANLAB), Leipziger Str. 44, Building 65, 39120 Magdeburg, Germany +49 3641 9390485 Taruntaak.kohla@gmail.com

4 Axel Krug (Prof.) MD Department. of Psychiatry and Psychotherapy, University of Bonn, D-53127 Bonn, Germany & Dept. of Psychiatry and Psychotherapy, Philipps Universität Marburg, Rudolf-Bultmann-Str. 8, 35039 Marburg, Germany axel.krug@ukbonn.de

5 Tilo Kircher (Prof.) MD Dept. of Psychiatry and Psychotherapy, Philipps Universität Marburg, Rudolf-Bultmann-Str. 8, 35039 Marburg, Germany +49 (0) 6421 58 65200 +49 (0) 6421 58 65197 kircher2@staff.uni-marburg.de

6 Igor Nenadić (Prof.) MD Dept. of Psychiatry and Psychotherapy, Philipps Universität Marburg, Rudolf-Bultmann-Str. 8, 35039 Marburg, Germany +49 (0) 6421 58 65002 +49 (0) 6421 58 68939 nenadic@staff.uni-marburg.de

7 Udo Dannlowski (Prof.). MD Institute for Translational Psychiatry, University of Münster, Münster, 48149, Germany +49 (0)251 / 83 - 56610 udo.dannlowski@uni-muenster.de

8 J. Paul Hamilton (Prof.) PhD Center for Social and Affective Neuroscience; Center for Medical Image Science and Visualization; Department of Biomedical and Clinical Sciences; Linköping University, Sweden +46 13 28 66 83 paul.hamilton@liu.se

9 Matthew D. Sacchet (Dr) PhD Center for Depression, Anxiety, and Stress Research, McLean Hospital, Harvard Medical School, Belmont, MA, USA +1 617 855 4437 msacchet@mclean.harvard.edu

10 Ian H. Gotlib (Prof.) PhD Department of Psychology, Bldg. 420, Jordan Hall, Stanford University, Stanford, CA 94305, USA + 1(650) 725-9216 ian.gotlib@stanford.edu

11 Henrik Walter (Prof.) MD Department of Psychiatry and Psychotherapy CCM, Charité – Universitätsmedizin Berlin, corporate member of Freie Universität Berlin, Berlin Institute of Health, Humboldt-Universität zu Berlin, Campus Charité Mitte, Charitéplatz 1, 10117, Berlin, Germany +49 30 450 517 141 +49 30 450 517 906 henrik.walter@charite.de

12 Thomas Frodl (Prof.) MD Department of Psychiatry and Psychotherapy, Otto von Guericke University Magdeburg, Leipzigerstr. 44, 39120 Magdeburg, Germany +49 391 6715029 thomas.frodl@med.ovgu.de

13 Simone Grimm (Prof.) PhD Department of Psychiatry and Psychotherapy, CBF, Charité Universitätsmedizin Berlin, 12203 Berlin, Germany +49 30 450 517 746 +49 30 450 517942 simone.grimm@charite.de

14 Ben J. Harrison (Prof.) PhD Melbourne Neuropsychiatry Centre, Department of Psychiatry, The University of Melbourne, Victoria, Australia +61 3 8344 1959 habj@unimelb.edu.au

15 Christian Robert Wolf (Prof.) MD Center for Psychosocial Medicine, Department of General Psychiatry, University of Heidelberg, Germany +49 6221 56-4405 +49 6221 56-4481 Christian.Wolf@med.uni-heidelberg.de

16 Sebastian Olbrich (Dr) MD Department of Psychiatry, Psychotherapy and Psychosomatic, University Zürich, Switzerland 8125  +41 44 296 7400 [sebastian.olbrich@pukzh.ch](mailto:sebastian.olbrich@pukzh.ch)

17 Guido van Wingen (Prof.) PhD Amsterdam UMC, University of Amsterdam, Department of Psychiatry, Amsterdam Neuroscience, Amsterdam, The Netherlands +31 20 8913523 guidovanwingen@gmail.com

18 Lukas Pezawas (Prof.) MD Department of Psychiatry and Psychotherapy, Medical University of Vienna, Austria +43 1 4020585 lukas.pezawas@meduniwien.ac.at

19 Gordon Parker (Prof.) MD School of Psychiatry, AGSM Building, University of New South Wales, Sydney, Australia +61-2-9385 7647 g.parker@unsw.edu.au

20 Matthew P. Hyett (Dr.) PhD School of Psychological Sciences, University of Western Australia, Perth, Australia +61 8 9266 5123 matthew.hyett@research.uwa.edu.au

21 Philipp G. Sämann (Dr.) MD Max Planck Institute of Psychiatry, Munich, Germany +49 (0) 89-30622-413 saemann@psych.mpg.de

22 Tim Hahn (Prof.) PhD Institute for Translational Psychiatry, University of Münster, Germany +49 251 83 56610 +49 251 83 57128 hahnt@wwu.de

23 Olaf Steinsträter (Dr.) MD Dept. of Psychiatry and Psychotherapy, Philipps Universität Marburg, Rudolf-Bultmann-Str. 8, 35039 Marburg, Germany steinstr@med.uni-marburg.de

24 Andreas Jansen (Prof.) PhD Department of Psychiatry and Psychotherapy & Marburg Center for Mind, Brain and Behavior - MCMBB, Philipps- Universität Marburg, Marburg, Germany +49(0)6421-28-23300 +49(0)6421-28-23301 jansena@med.uni-marburg.de

25 Dilara Yuksel (Dr) PhD Center for Health Sciences, SRI International, 333 Ravenswood Avenue, Menlo Park, CA, USA. dilara.yuksel@sri.co

26 Robin Kämpe (Mr.) MS Center for Social and Affective Neuroscience; Center for Medical Image Science and Visualization; Department of Biomedical and Clinical Sciences; Linköping University +46 13 28 19 74 robin.kampe@liu.se

27 Christopher G. Davey (Prof.) MD PhD Department of Psychiatry, The University of Melbourne, Victoria, Australia c.davey@unimelb.edu.au

28 Bernhard Meyer (Prof.) PhD Department of Psychiatry and Psychotherapy, Medical University of Vienna, Austria bernhard.meyer@edupression.com

29 Lucie Bartova (Prof.) MD PhD Department of Psychiatry and Psychotherapy, Medical University of Vienna, Austria lucie.bartova@meduniwien.ac.at

30 Ilona Croy (Prof.) PhD Department of Psychology, Friedrich Schiller University Jena, Germany & Department of Psychotherapy and Psychosomatic Medicine, TU Dresden, Germany +49 3641 945140 +49 3641 945142 ilona.croy@uni-jena.de

* 31 Martin Walter (Prof.) MD PhD Department of Psychiatry and Psychotherapy, Jena University Hospital, Philosophenweg 3, 07743 Jena, Germany & Clinical Affective Neuroimaging Laboratory (CANLAB), Leipziger Str. 44, Building 65, 39120 Magdeburg, Germany & Leibniz Institute for Neurobiology, Brenneckestr. 6, 39118 Magdeburg, Germany & Department of Psychiatry and Psychotherapy, University Tuebingen, Calwerstraße 14, 72076 Tuebingen, Germany +49 3641 9390101 Martin.Walter@med.uni-jena.de

**† *** 32 Gerd Wagner (PD Dr.) PhD Department of Psychiatry and Psychotherapy, Jena University Hospital, Philosophenweg 3, 07743 Jena, Germany +49 36419390421 wagner.gerd@uni-jena.de

***Equal contribution**

**†Corresponding Author:**

Priv.-Doz. Dr. Gerd Wagner (wagner.gerd@uni-jena.de), Department of Psychiatry and Psychotherapy, Jena University Hospital, Philosophenweg 3, 07743 Jena, Germany

**Methods**

***Sample and site information***

The raw NIFTI files of the resting state fMRI and anatomical data, demographic and clinical information were provided to the consortium, and the present analyses have been approved by the PsyMRI members. T1 images were defaced and all identifications were anonymized before further image processing.

We applied conservative exclusion criteria to control for site and demographic variation (see PRISMA flow diagram in supplementary Figure 1). We restricted our analysis to datasets acquired with the 3 Tesla scanners ^1^. The scanner information and further acquisition details for each site can be found in supplementary Table 1. We also limited the age band to the adults range (18 and 65 years) to reduce the variance in FC due to potential confounding neurodegenerative disorders in the elderlies or comorbid developmental conditions in adolescents ^2^.

Our further exclusion criteria at the subject level were: subject drop-outs reported by study centers, incomplete or missing neuroimaging, and demographic data, excessive head motion (mean of frame-wise displacement (meanFD) > 0.55) ^3^. We finally included 1082 participants in the key analysis (MDD: 606, HC: 476) as reported in table 1. There were no significant differences regarding age and gender between MDD and HC groups.

Depression severity across included studies were acquired using different standard depression scales. The total scores of these questionnaires from different centers were converted to five depression severity categories (supplementary table 3): remitted, mild, moderate, severe and very severe ^4,5^. The average scores of site-specific questionnaires are reported in supplementary table 4.

## ***Preprocessing of fMRI data***

Prior to preprocessing, all MR images were visually inspected and the origin was set manually to anterior commissure to improve co-registration.

For preprocessing, we utilized DPARSF(V4.3) from DPABI toolbox ^6^ developed based on SPM12 ([www.fil.ion.ucl.ac.uk/spm/](http://www.fil.ion.ucl.ac.uk/spm/)) in MATLAB – Mathworks (2019a) ( [www.mathworks.com](http://www.mathworks.com/)). In the first step, we discarded the first 10 volumes to obtain steady-state tissue magnetization, applied slice-timing correction, and realigned the images using rigid-body linear transformation. Next, each T1-weighted image was co-registered to the mean realigned image. Subsequently, the native T1 images were transformed to MNI space by Diffeomorphic Anatomical Registration Through Exponentiated Lie algebra (DARTEL) after segmentation into gray matter (GM), white matter (WM) and cerebrospinal fluid (CSF) ^7^. The resulting flow field was then applied to the functional images to gain a spatially normalized fMRI dataset.

We applied the Friston 24-parameter method to regress out head motion effects and additionally calculated mean framewise displacement ^8^ to exclude subjects with gross head motion (mean FD > 0.55) ^3^. The FD was also used as a covariate in the statistical model to further control for motion-related artifacts ^9^. Furthermore, linear and quadratic trends were removed and bandpass filtering (0.01–0.1 Hz) was subsequently performed after regressing out signal from white matter and CSF. The functional images were smoothed with a 6 mm (FWHM) filter.

**Results**

***Replication of the previous mega-analysis***^10^

To replicate the DMN finding of the previous study ^10^, we used the same methodology as described in the paper. Thus, we extracted the time courses of 160 region of interests (ROIs) based on Dosenbach atlas (6 mm sphere) and calculated a correlation coefficient matrix for all 160 nodes and applied Fisher-Z transformation. The same DMN nodes were extracted as used in the previous study ^10^. Comparing averaged FCs from DMN nodes, a non-significant difference was found between MDD patients and healthy controls (p-value = 0.050).

***Functional connectivity within DMN nodes***

To compare pair-wise FCs within DMN, we selected 91 DMN nodes and compared each of the 4095 FCs between MDD patients and healthy controls. We did not find any significant differences in FCs within DMN between both groups at the FDR adjusted p-level of < 0.05.

***Forest plot for averaged FCs within DMN, SMN, SN***

To compare the findings for averaged FCs between sites, we used linear fixed effect model having gender, age and mean FD as fixed variables for each site showing mean differences between MDD patients and healthy controls after adjusting for the mentioned covariates (supplementary figure 4, 5 and 6).

**Association between network segregation and age**

We compared the slopes of the association between age and network segregation measure between healthy controls and MDD patients. As depicted in the supplementary figure 7, MDD patients exhibited a significantly greater negative slope than healthy controls (p-value = 0.035).

**Reference:**

1. Friedman, L., Glover, G. H., Krenz, D. & Magnotta, V. Reducing inter-scanner variability of activation in a multicenter fMRI study: Role of smoothness equalization. *NeuroImage* **32**, 1656–1668 (2006).

2. Wierenga, L. M. *et al.* Unraveling age, puberty and testosterone effects on subcortical brain development across adolescence. *Psychoneuroendocrinology* **91**, 105–114 (2018).

3. Satterthwaite, T. D. *et al.* An improved framework for confound regression and filtering for control of motion artifact in the preprocessing of resting-state functional connectivity data. *NeuroImage* **64**, 240–256 (2013).

4. Leucht, S., Fennema, H., Engel, R. R., Kaspers-Janssen, M. & Szegedi, A. Translating the HAM-D into the MADRS and vice versa with equipercentile linking. *J. Affect. Disord.* **226**, 326–331 (2018).

5. Rush, A. J. *et al.* An evaluation of the quick inventory of depressive symptomatology and the hamilton rating scale for depression: a sequenced treatment alternatives to relieve depression trial report. *Biol. Psychiatry* **59**, 493–501 (2006).

6. Yan, C.-G., Wang, X.-D., Zuo, X.-N. & Zang, Y.-F. DPABI: Data Processing & Analysis for (Resting-State) Brain Imaging. *Neuroinformatics* **14**, 339–351 (2016).

7. Ashburner, J. & Friston, K. J. Diffeomorphic registration using geodesic shooting and Gauss–Newton optimisation. *NeuroImage* **55**, 954–967 (2011).

8. Power, J. D., Barnes, K. A., Snyder, A. Z., Schlaggar, B. L. & Petersen, S. E. Spurious but systematic correlations in functional connectivity MRI networks arise from subject motion. *NeuroImage* **59**, 2142–2154 (2012).

9. Spisák, T. *et al.* Voxel-Wise Motion Artifacts in Population-Level Whole-Brain Connectivity Analysis of Resting-State fMRI. *PLOS ONE* **9**, e104947 (2014).

10. Yan, C.-G. *et al.* Reduced default mode network functional connectivity in patients with recurrent major depressive disorder. *Proc. Natl. Acad. Sci.* **116**, 9078–9083 (2019).

**Supplemental table 1: Rs-fMRI specific information for each PsyMRI site**

This table depicts the scanner information including scanner manufacturer, magnetic field, scanner model and acquisition information, and the number of participants in each group that each center contributed to the PsyMRI consortium.

| Scanner brand | Siemens | | | | | | | | | Philips | | | GE | |
| --- | --- | --- | --- | --- | --- | --- | --- | --- | --- | --- | --- | --- | --- | --- |
| Magnetic field | 3T | | | | | | | | | | | | | |
| Scanner model | Trio Tim | | | | Allegra | Magnetom Trio | | | Prisma | Achieva | | | Discovery 750 | |
| City/site | Berlin/Charite | Marburg_DFG | Marburg_FOR2017 | Wien | Ulm/Heidelberg | Jena | Leipzig | Magdeburg | Linköping | Sydney | Dublin | | Stanford_qMRI | Stanford_MIG |
| Time points | 210 | 110 | 237 | 180 | 180 | 240 | 275 | 488 | 583 | 186 | 210 | 180 | 240 | 200 |
| Number of slices | 37 | 40 | 33 | 20 | 33 | 45 | 40 | 26 | 48 | 33 | 40 | 39 | 42 | 42 |
| TR | 2.3 | 2.5 | 2 | 2 | 2 | 2.52 | 2.4 | 1.25 | 1.3 | 2 | 2 | 2 | 2 | 2 |
| Voxel resolution | 3.0 x 3.0 x 3.29 | 3.5 x 3.5 x 3.85 | 3.28 x 3.28 x 4.18 | 2.18 x 2.18 x 5.0 | 3.0 x 3.0 x 3.60 | 2.5 x 2.5 x 3.125 | 2.30 x 2.30 x 3.30 | 5.0 x 5.0 x 5.0 | 3.0 x 3.0 x 3.0 | 1.87 x 1.87 x 3.20 | 3.0 x 3.0 x 3.30 | 3.5 x 3.5 x 3.85 | 2.9 x 2.9 x 2.9 | 2.90 x 2.90 x 2.89 |
| Illumination | Off | 50% | Off | Off | Off | On | Off | Off |  | Off | On/Off | Off | On | On |
| Eyes open/close | Open | Close | Close | Close | Close | Close | Close | Close | Open | Close | Open/Close | Close | Close | Close |
| Before/after task | Before | Before | Before | Before | Before | Before | Before | Before |  | Before | Before | Before |  | After |
| Number of HC | 18 | 97 | 91 | 44 | 14 | 51 | 17 | 22 | 23 | 21 | 14 | 38 | 37 | 18 |
| Number of MDD | 23 | 92 | 140 | 80 | 34 | 51 | 20 | 21 | 53 | 35 | 46 | 0 | 37 | 18 |

**Supplemental table 2:** **Demographic characteristics of each PsyMRI center included in the study**

|  | **Berlin - Charite** | | **Sydney** | | **Dublin** | | **Marburg_DFG** | | **Marburg_FOR2107** | |
| --- | --- | --- | --- | --- | --- | --- | --- | --- | --- | --- |
|  | **Controls** | **Patients** | **Controls** | **Patients** | **Controls** | **Patients** | **Controls** | **Patients** | **Controls** | **Patients** |
|  | **(N=18)** | **(N=19)** | **(N=15)** | **(N=28)** | **(N=47)** | **(N=42)** | **(N=95)** | **(N=90)** | **(N=80)** | **(N=118)** |
| **gender** |  |  |  |  |  |  |  |  |  |  |
| Male | 18.0 (100%) | 5.00 (26.3%) | 5.00 (33.3%) | 12.0 (42.9%) | 15.0 (31.9%) | 17.0 (40.5%) | 57.0 (60.0%) | 49.0 (54.4%) | 28.0 (35.0%) | 44.0 (37.3%) |
| Female | 0 (0%) | 14.0 (73.7%) | 10.0 (66.7%) | 16.0 (57.1%) | 32.0 (68.1%) | 25.0 (59.5%) | 38.0 (40.0%) | 41.0 (45.6%) | 52.0 (65.0%) | 74.0 (62.7%) |
| **age** |  |  |  |  |  |  |  |  |  |  |
| Mean (SD) | 28.3 (4.69) | 44.1 (10.6) | 36.7 (11.2) | 39.1 (11.1) | 39.4 (14.3) | 35.3 (11.5) | 31.5 (9.57) | 35.7 (11.0) | 34.9 (10.6) | 38.2 (13.5) |
| Median [Min, Max] | 29.0 [21.0, 37.0] | 46.0 [26.0, 59.0] | 35.0 [22.0, 62.0] | 40.0 [19.0, 60.0] | 38.0 [18.0, 65.0] | 33.5 [20.0, 60.0] | 29.0 [19.0, 57.0] | 36.5 [18.0, 55.0] | 31.0 [20.0, 62.0] | 36.5 [18.0, 65.0] |
|  | **Leipzig** | | **Linköping** | | **Magdeburg** | | **Stanford_MIG** | |  |  |
|  | **Controls** | **Patients** | **Controls** | **Patients** | **Controls** | **Patients** | **Controls** | **Patients** |  |  |
|  | **(N=17)** | **(N=18)** | **(N=23)** | **(N=53)** | **(N=21)** | **(N=21)** | **(N=18)** | **(N=18)** |  |  |
| **gender** |  |  |  |  |  |  |  |  |  |  |
| Male | 7.00 (41.2%) | 8.00 (44.4%) | 6.00 (26.1%) | 15.0 (28.3%) | 13.0 (61.9%) | 13.0 (61.9%) | 6.00 (33.3%) | 6.00 (33.3%) |  |  |
| Female | 10.0 (58.8%) | 10.0 (55.6%) | 17.0 (73.9%) | 38.0 (71.7%) | 8.00 (38.1%) | 8.00 (38.1%) | 12.0 (66.7%) | 12.0 (66.7%) |  |  |
| **age** |  |  |  |  |  |  |  |  |  |  |
| Mean (SD) | 37.6 (11.5) | 37.3 (11.8) | 31.9 (11.4) | 36.5 (13.8) | 35.0 (6.00) | 37.9 (11.4) | 25.9 (3.16) | 25.8 (3.40) |  |  |
| Median [Min, Max] | 35.0 [19.0, 59.0] | 37.0 [22.0, 62.0] | 27.0 [19.0, 54.0] | 33.0 [18.0, 65.0] | 35.0 [25.0, 44.0] | 38.0 [20.0, 57.0] | 25.2 [20.9, 31.4] | 25.5 [20.4, 31.8] |  |  |
|  | **Jena** | | **Ulm/Heidelberg** | | **Wien** | | **Stanford_qMRI** | |  |  |
|  | **Controls** | **Patients** | **Controls** | **Patients** | **Controls** | **Patients** | **Controls** | **Patients** |  |  |
|  | **(N=49)** | **(N=49)** | **(N=13)** | **(N=33)** | **(N=44)** | **(N=80)** | **(N=36)** | **(N=37)** |  |  |
| **gender** |  |  |  |  |  |  |  |  |  |  |
| Male | 14.0 (28.6%) | 13.0 (26.5%) | 3.00 (23.1%) | 10.0 (30.3%) | 22.0 (50.0%) | 32.0 (40.0%) | 12.0 (33.3%) | 14.0 (37.8%) |  |  |
| Female | 35.0 (71.4%) | 36.0 (73.5%) | 10.0 (76.9%) | 23.0 (69.7%) | 22.0 (50.0%) | 48.0 (60.0%) | 24.0 (66.7%) | 23.0 (62.2%) |  |  |
| **age** |  |  |  |  |  |  |  |  |  |  |
| Mean (SD) | 37.0 (11.6) | 36.8 (12.1) | 26.2 (11.1) | 39.6 (9.70) | 25.3 (3.27) | 28.0 (6.05) | 36.0 (12.1) | 35.9 (12.4) |  |  |
| Median [Min, Max] | 34.0 [20.0, 59.0] | 33.0 [20.0, 58.0] | 21.0 [19.0, 59.0] | 41.0 [20.0, 57.0] | 25.0 [19.0, 33.0] | 26.0 [19.0, 46.0] | 33.4 [19.1, 58.4] | 32.0 [20.8, 56.5] |  |  |

**Supplemental table 3:** **Demographic characteristics of patients grouped by depression severity**

|  | Remitted | Mild | Moderate | Severe | Very sever | n.a. | Total |
| --- | --- | --- | --- | --- | --- | --- | --- |
|  | (N=141) | (N=102) | (N=201) | (N=101) | (N=39) | (N=22) | (N=606) |
| age |  |  |  |  |  |  |  |
| Mean (SD) | 33.8 (12.3) | 36.5 (12.1) | 35.7 (11.5) | 37.6 (11.8) | 39.0 (11.9) | 31.6 (11.3) | 35.8 (11.9) |
| Median [Min, Max] | 28.2 [20.0, 65.0] | 35.0 [18.0, 65.0] | 33.0 [18.0, 62.0] | 38.0 [19.0, 60.0] | 39.0 [19.0, 60.0] | 28.5 [18.0, 54.0] | 32.6 [18.0, 65.0] |
| gender |  |  |  |  |  |  |  |
| Female | 83.0 (58.9%) | 63.0 (61.8%) | 127 (63.2%) | 60.0 (59.4%) | 21.0 (53.8%) | 14.0 (63.6%) | 368 (60.7%) |
| Male | 58.0 (41.1%) | 39.0 (38.2%) | 74.0 (36.8%) | 41.0 (40.6%) | 18.0 (46.2%) | 8.00 (36.4%) | 238 (39.3%) |

**Supplemental table 4: Depression scales and the average total score provided by each center**

| **Site** | **Questionnaire** | **Female** | **Male** | **Total** |
| --- | --- | --- | --- | --- |
| **Berlin** | **HRDS_24** | **(N=14)** | **(N=6)** | **(N=20)** |
|  | Mean (SD) | 23.2 (6.42) | 22.0 (2.19) | 22.9 (5.46) |
|  | Median [Min, Max] | 21.5 [16.0, 38.0] | 22.5 [19.0, 25.0] | 22.0 [16.0, 38.0] |
| **Sydney** | **QIDS** | **(N=16)** | **(N=12)** | **(N=28)** |
|  | Mean (SD) | 14.8 (4.02) | 18.3 (3.58) | 16.3 (4.16) |
|  | Median [Min, Max] | 13.0 [11.0, 24.0] | 17.5 [14.0, 25.0] | 16.0 [11.0, 25.0] |
| **Dublin** | **HRDS_17** | **(N=25)** | **(N=17)** | **(N=42)** |
|  | Mean (SD) | 22.4 (5.72) | 22.1 (5.89) | 22.3 (5.72) |
|  | Median [Min, Max] | 23.0 [6.00, 36.0] | 23.0 [8.00, 32.0] | 23.0 [6.00, 36.0] |
| **Marburg_DFG** | **HRDS_17** | **(N=41)** | **(N=49)** | **(N=90)** |
|  | Mean (SD) | 15.7 (5.35) | 15.1 (7.10) | 15.3 (6.40) |
|  | Median [Min, Max] | 16.0 [4.00, 26.0] | 15.0 [1.00, 30.0] | 16.0 [1.00, 30.0] |
| **Marburg_FOR2017** | **HRDS_17** | **(N=74)** | **(N=44)** | **(N=118)** |
|  | Mean (SD) | 8.57 (6.28) | 8.50 (7.54) | 8.54 (6.75) |
|  | Median [Min, Max] | 8.00 [0, 23.0] | 6.50 [0, 34.0] | 8.00 [0, 34.0] |
| **Ulm/Heidelberg** | **HRDS_21** | **(N=23)** | **(N=10)** | **(N=33)** |
|  | Mean (SD) | 18.8 (7.55) | 19.1 (7.69) | 18.9 (7.47) |
|  | Median [Min, Max] | 20.0 [0, 31.0] | 19.5 [1.00, 27.0] | 20.0 [0, 31.0] |
| **Jena** | **HRDS_21** | **(N=36)** | **(N=13)** | **(N=49)** |
|  | Mean (SD) | 20.6 (10.9) | 19.8 (8.78) | 20.4 (10.3) |
|  | Median [Min, Max] | 20.0 [4.00, 43.0] | 18.0 [6.00, 37.0] | 19.5 [4.00, 43.0] |
| **Leipzig** | **HRDS_21** | **(N=10)** | **(N=8)** | **(N=18)** |
|  | Mean (SD) | 23.9 (2.24) | 22.2 (2.78) | 23.1 (2.56) |
|  | Median [Min, Max] | 24.0 [21.0, 28.5] | 21.8 [18.5, 27.0] | 23.5 [18.5, 28.5] |
| **Linköping** | **MADRS** | **(N=38)** | **(N=15)** | **(N=53)** |
|  | Mean (SD) | 27.7 (4.93) | 27.8 (7.19) | 27.7 (5.59) |
|  | Median [Min, Max] | 28.0 [16.0, 35.0] | 27.0 [14.0, 41.0] | 28.0 [14.0, 41.0] |
| **Magdeburg** | **HRDS_17** | **(N=8)** | **(N=13)** | **(N=21)** |
|  | Mean (SD) | 15.8 (4.23) | 16.2 (5.09) | 16.0 (4.67) |
|  | Median [Min, Max] | 15.5 [8.00, 20.0] | 16.0 [8.00, 23.0] | 16.0 [8.00, 23.0] |
| **Stanford_MIG** | **HRDS_17** | **(N=12)** | **(N=6)** | **(N=18)** |
|  | Mean (SD) | 15.3 (3.65) | 11.7 (4.93) | 14.1 (4.35) |
|  | Median [Min, Max] | 14.5 [11.0, 24.0] | 13.5 [5.00, 16.0] | 14.0 [5.00, 24.0] |
| **Stanford_qMRI** | **HRDS_17** | **(N=23)** | **(N=14)** | **(N=37)** |
|  | Mean (SD) | 16.4 (4.70) | 14.5 (8.24) | 15.7 (6.24) |
|  | Median [Min, Max] | 16.0 [3.00, 23.0] | 13.0 [3.00, 30.0] | 16.0 [3.00, 30.0] |
| **Vienna** | **HRDS_17** | **(N=48)** | **(N=32)** | **(N=80)** |
|  | Mean (SD) | 6.79 (8.18) | 7.88 (8.92) | 7.23 (8.44) |
|  | Median [Min, Max] | 3.00 [0, 25.0] | 3.00 [0, 26.0] | 3.00 [0, 26.0] |

**Supplemental table 5:** **Demographic characteristics of patients treated with psychopharmacological drugs vs. unmedicated patients.**

|  | **medicated** | **unmedicated** | **Total** | **P-value** |
| --- | --- | --- | --- | --- |
|  | **(N=370)** | **(N=211)** | **(N=584)** |  |
| **age** |  |  |  | t-test |
| Mean (SD) | 37.6 (12.2) | 33.1 (10.8) | 35.9 (11.9) | <0.001 |
| **gender** |  |  |  | Chi-Square test |
| Female | 230 (62.2%) | 121 (57.3%) | 351 (60.6%) | 0.2922 |
| Male | 140 (37.8%) | 90.0 (42.7%) | 230 (39.4%) |  |

Medication not reported (N=23)

**Supplemental table 6: Averaged FCs within each network and statistical comparisons between medicated vs. unmedicated patients.**

| **Network** | **Adjusted mean medicated** | **Adjusted mean unmedicated** | **t-value** | **Cohen's d** | **Uncorrected P-value** |
| --- | --- | --- | --- | --- | --- |
| FPN | -0.096 | 0.201 | 2.962 | 0.264 | 0.003* |
| DMN | -0.127 | 0.172 | 3.004 | 0.264 | 0.002* |
| DAN | -0.081 | 0.129 | 2.186 | 0.185 | 0.0298 |
| LN | -0.080 | 0.072 | 1.529 | 0.130 | n.s |
| SN | -0.155 | 0.189 | 3.482 | 0.296 | 0.0005* |
| SMN | -0.189 | 0.138 | 3.364 | 0.286 | 0.0007* |
| VN | -0.112 | 0.118 | 2.348 | 0.198 | 0.018 |

* Survives Bonferroni correction

**Supplemental table 7: Segregation of each brain network and statistical comparisons between medicated vs. unmedicated patients.**

| **Network** | **Adjusted mean medicated** | **Adjusted mean unmedicated** | **t-value** | **Cohen's d** | **Uncorrected P-value** |
| --- | --- | --- | --- | --- | --- |
| FPN | 0.032 | -0.105 | -1.531 | -0.149 | n.s |
| DMN | 0.034 | -0.188 | -2.466 | -0.219 | 0.012 |
| DAN | 0.107 | -0.156 | -2.811 | -0.237 | 0.004* |
| LN | -0.027 | -0.255 | -2.302 | -0.194 | 0.020 |
| SN | 0.015 | -0.090 | -1.155 | -0.104 | n.s |
| SMN | -0.099 | -0.120 | -0.241 | -0.021 | n.s |
| VN | 0.066 | -0.135 | -2.117 | -0.181 | 0.033 |

* Survives Bonferroni correction

**Supplemental figure 1: PRISMA flow diagram.**

This flow-diagram illustrates the exclusion and inclusion criteria of the current study and the number of subjects excluded from the study. At the end we included 606 MDD patients and 476 healthy controls and in total 1082 participants.

**Supplemental figure 2: The bar-plot of the sample size contributed from each center.**

This bar-plot visualize the sample size each included center contributed to the PsyMRI consortium after applying inclusion criteria

**Supplemental figure 3: Forest plot based on the mean FC values within DMN over sites.**

The forest plot illustrates the mean differences of averaged FCs within DMN for each site adjusted for age, gender and mean FD.

**Supplemental figure 4: Forest plot based on the mean FC values within SMN across centers.**

The forest plot illustrates the mean differences of averaged FCs within SMN for each site adjusted for age, gender and mean FD.

**Supplemental figure 5: Forest plot based on the averaged FC values within SN across centers.**

The forest plot illustrates the mean differences of averaged FCs within SN for each site adjusted for age, gender and mean FD.

**Supplemental figure 6: Significant group differences in FCs between 400 nodes of the seven cortical networks projected on the brain map.**

In this figure each color in the brain map represents a network. The grey lines show lower FC in patients with MDD compared to HCs. The FC differences between 400 nodes were obtained from LMER with age, gender, meanFD as fixed variable and site as random variable. The false discovery rate (FDR) was used to correct for multiple comparisons (p-value < 0.05).

**Supplemental figure 7: Significant group differences in FCs within and between networks over 400 nodes based on the meta-analysis.**

In this figure each color in the circle represents a network. The blue color is showing lower FC in patients with MDD comparing to healthy controls. We used the false discovery rate (FDR) (p-value < 0.05) to correct for multiple comparisons.

**Supplemental figure 8: Linear trends of the association between SMN segregation and age for MDD patients and controls.**

This figure is showing the linear trend of SMN segregation and age for patients (red line) and controls (turquoise line)
