# Supplementary material for: Altered resting-state functional connectome in major depressive disorder: a mega-analysis from the PsyMRI consortium
Source: Transl Psychiatry. 2021 Oct 7;11:511. doi: 10.1038/s41398-021-01619-w (PMC8497531; doi:10.1038/s41398-021-01619-w)
Supplement: Supplementary file 2 — Supplemental Figure 1 [file 41398_2021_1619_MOESM2_ESM.pdf]

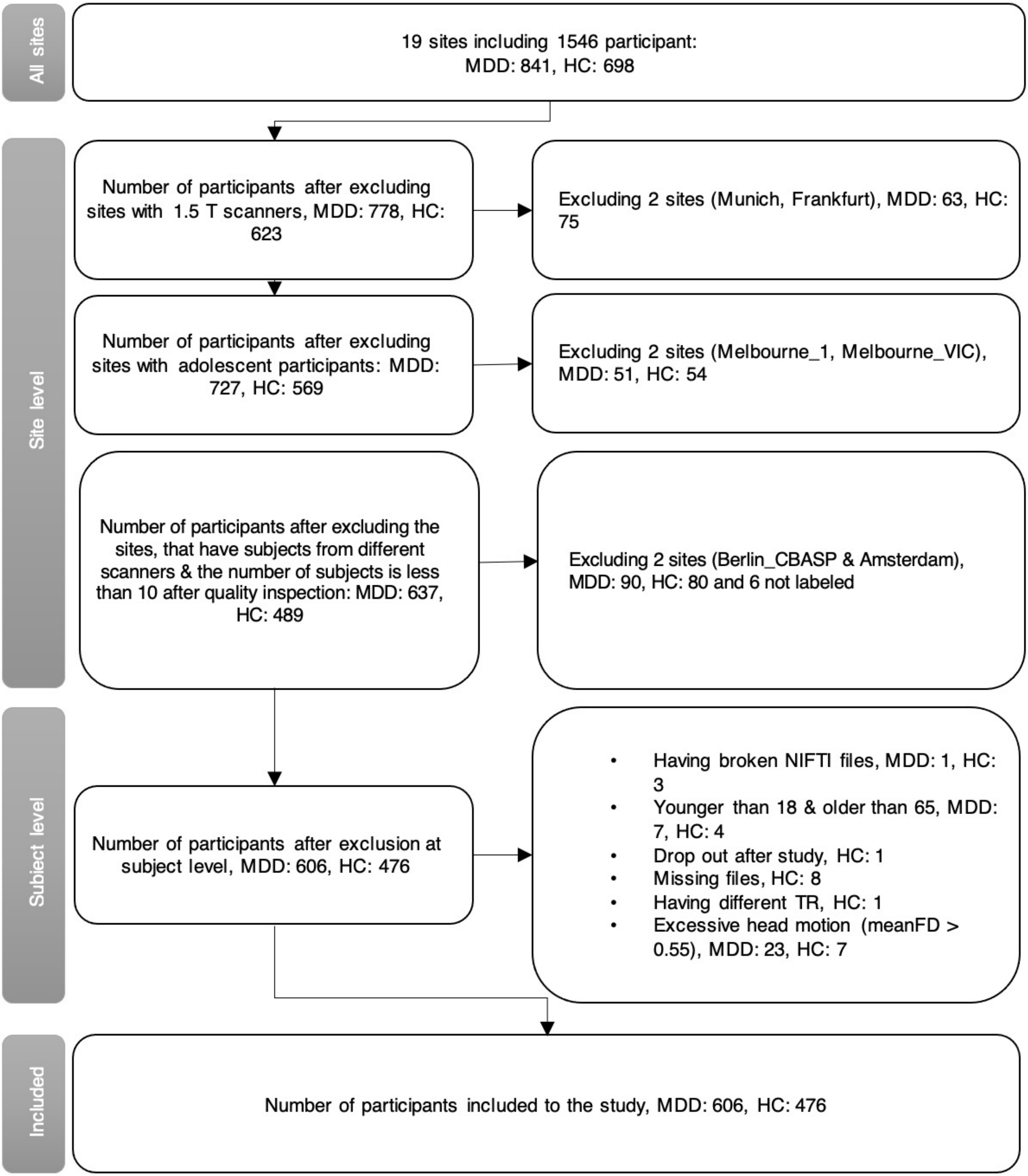
All sites
19 sites including 1546 participant: MDD: 841, HC: 698
Site level
Number of participants after excluding sites with 1.5 T scanners, MDD: 778, HC: 623
Excluding 2 sites (Munich, Frankfurt), MDD: 63, HC: 75
Number of participants after excluding sites with adolescent participants: MDD: 727, HC: 569
Excluding 2 sites (Melbourne_1, Melbourne_VIC), MDD: 51, HC: 54
Number of participants after excluding the sites, that have subjects from different scanners & the number of subjects is less than 10 after quality inspection: MDD: 637, HC: 489
Excluding 2 sites (Berlin_CBASP & Amsterdam), MDD: 90, HC: 80 and 6 not labeled
Subject level
Number of participants after exclusion at subject level, MDD: 606, HC: 476
• Having broken NIFTI files, MDD: 1, HC: 3
• Younger than 18 & older than 65, MDD: 7, HC: 4
• Drop out after study, HC: 1
• Missing files, HC: 8
• Having different TR, HC: 1
• Excessive head motion (meanFD > 0.55), MDD: 23, HC: 7
Included
Number of participants included to the study, MDD: 606, HC: 476
