# Supplementary figures and images for: Altered resting-state functional connectome in major depressive disorder: a mega-analysis from the PsyMRI consortium
Source: Transl Psychiatry. 2021 Oct 7;11:511. doi: 10.1038/s41398-021-01619-w (PMC8497531; doi:10.1038/s41398-021-01619-w)

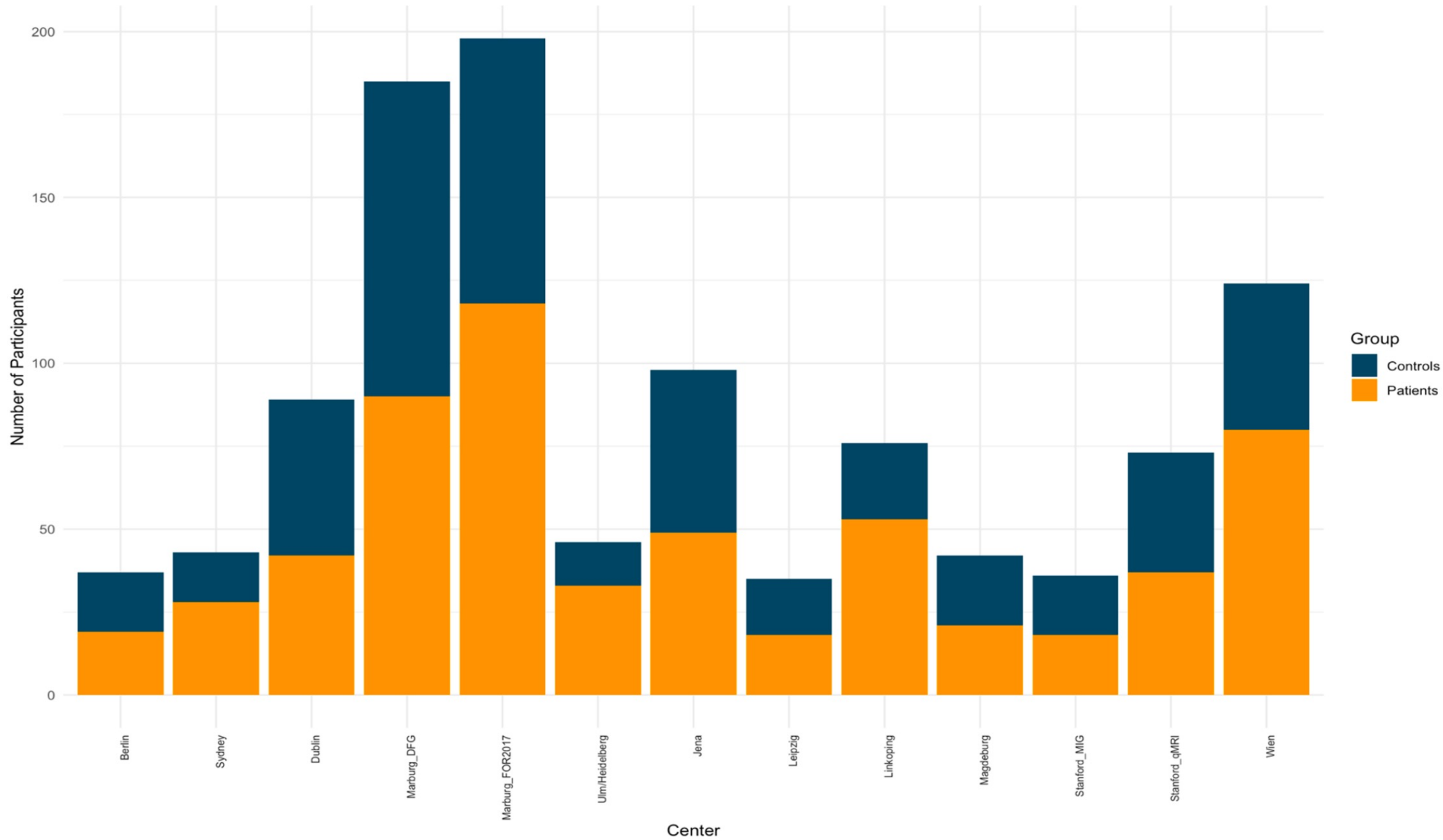

Supplement: Supplementary file 3 — Supplemental Figure 2 [file 41398_2021_1619_MOESM3_ESM.pdf]

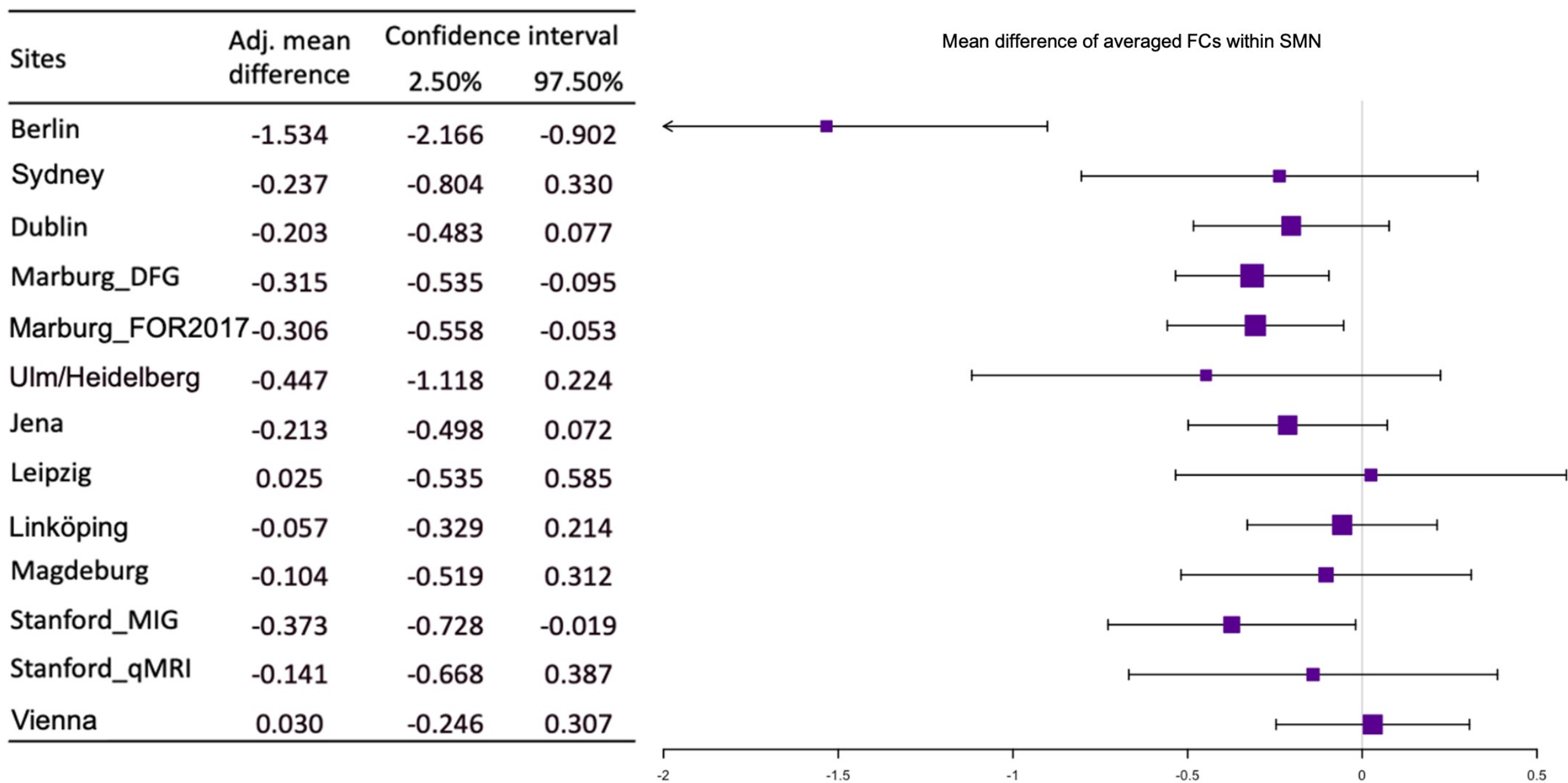

Supplement: Supplementary file 5 — Supplemental Figure 4 [file 41398_2021_1619_MOESM5_ESM.pdf]

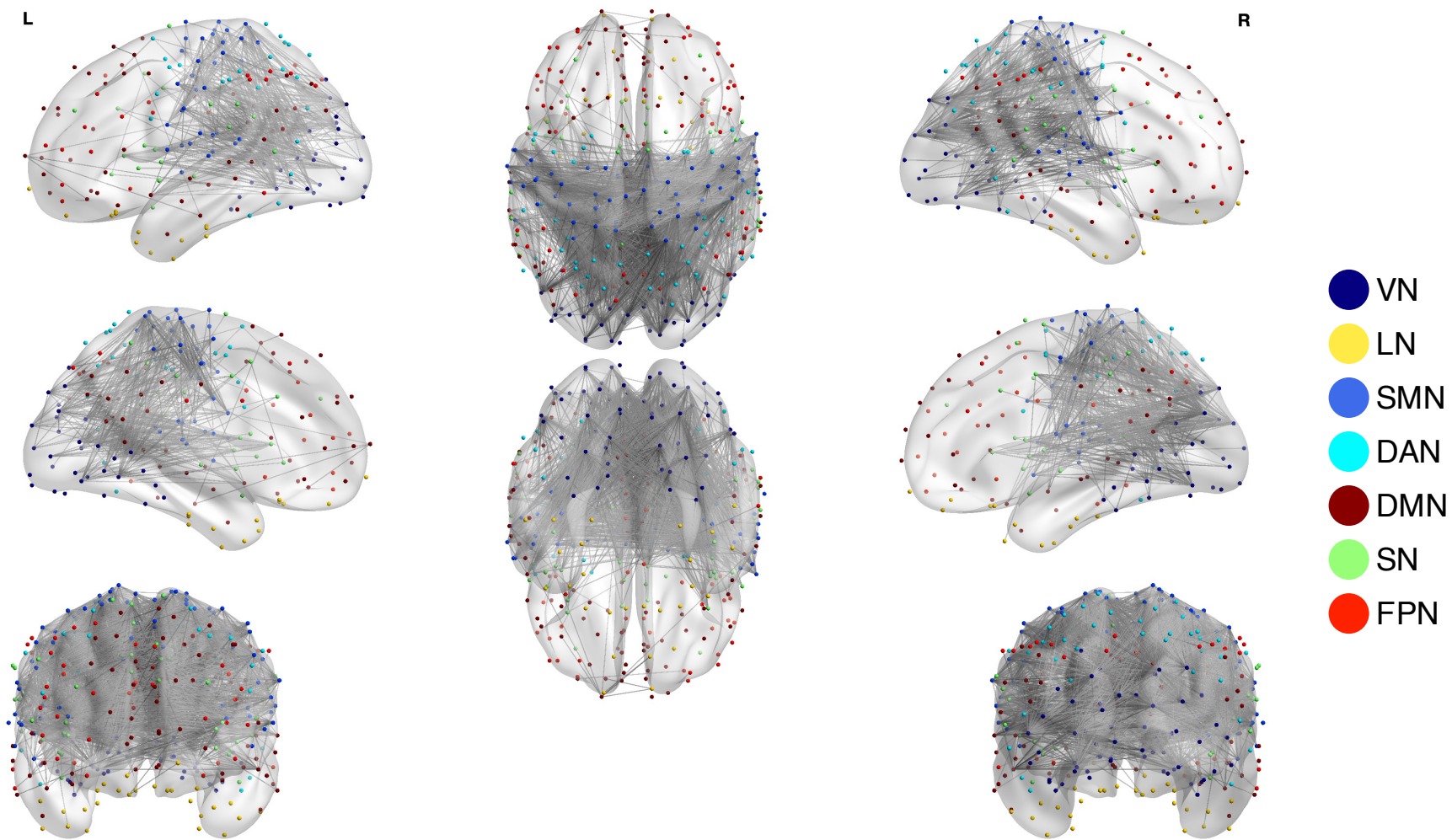

Supplement: Supplementary file 7 — Supplemental Figure 6 [file 41398_2021_1619_MOESM7_ESM.pdf]

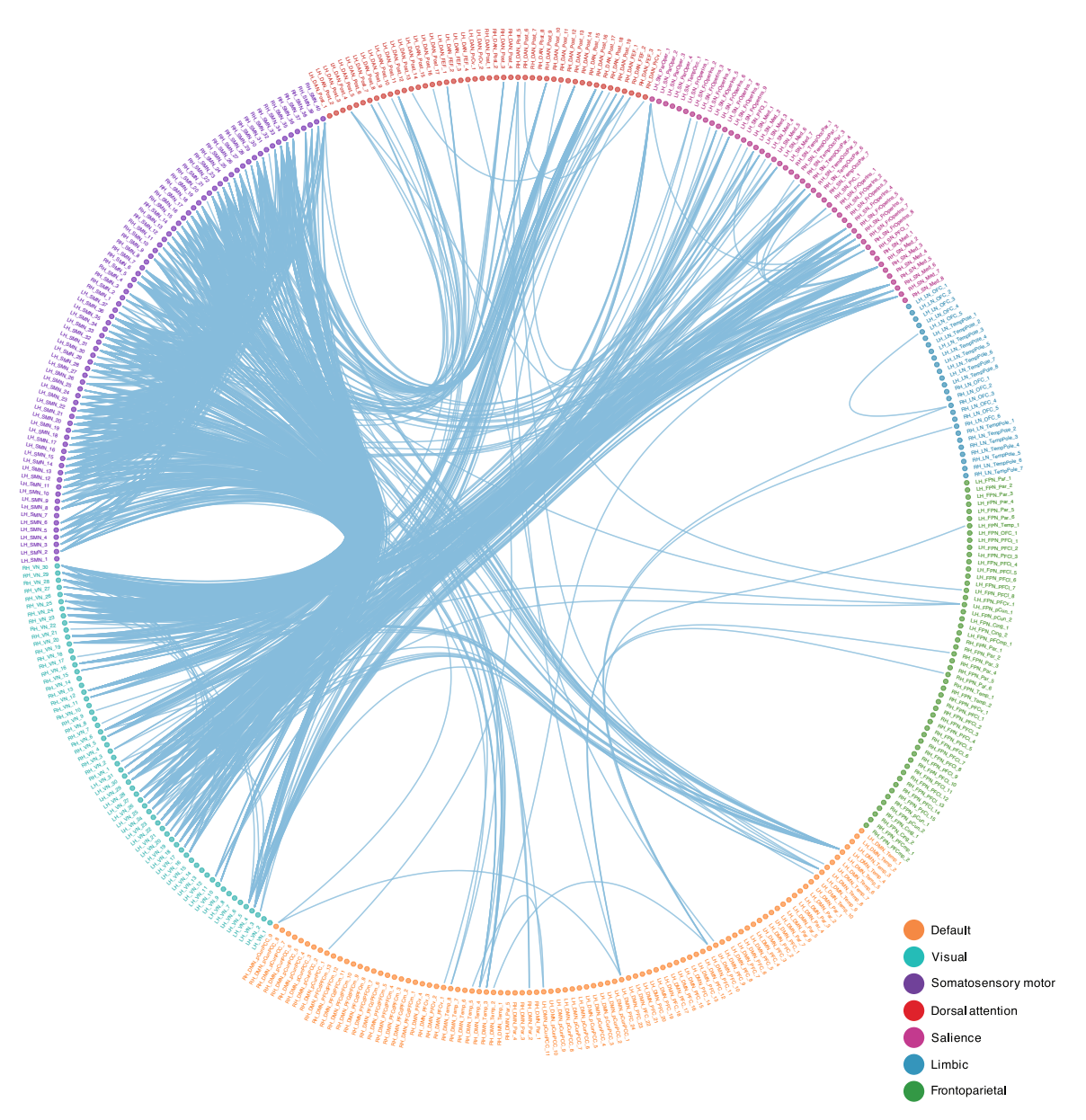

Supplement: Supplementary file 8 — Supplemental Figure 7 [file 41398_2021_1619_MOESM8_ESM.pdf]

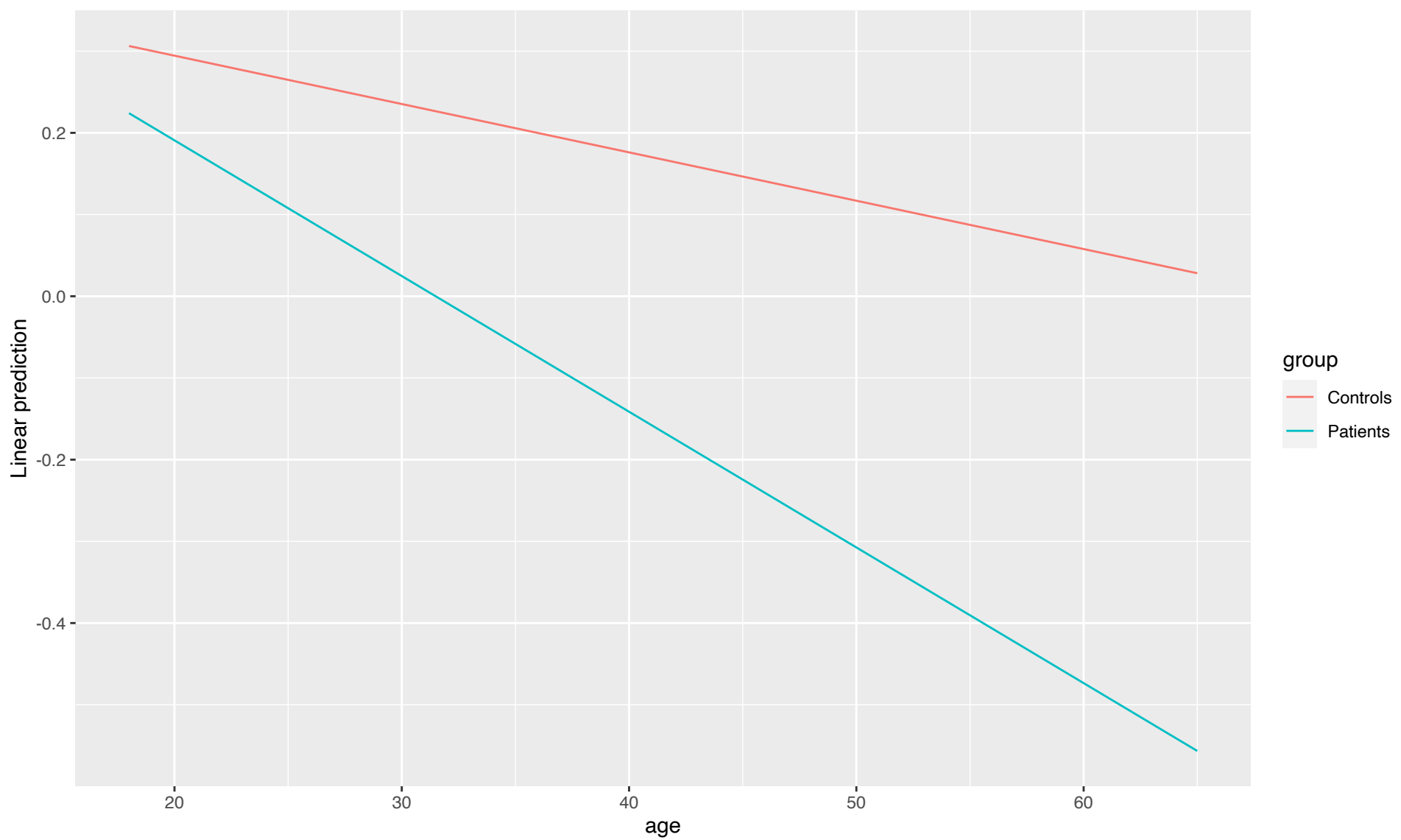

Supplement: Supplementary file 9 — Supplemental Figure 8 [file 41398_2021_1619_MOESM9_ESM.pdf]
