# Supplementary material for: Altered resting-state functional connectome in major depressive disorder: a mega-analysis from the PsyMRI consortium
Source: Transl Psychiatry. 2021 Oct 7;11:511. doi: 10.1038/s41398-021-01619-w (PMC8497531; doi:10.1038/s41398-021-01619-w)

| Sites | Adj. mean difference | Confidence interval | |
| --- | --- | --- | --- |
| | | 2.50% | 97.50% |
| Berlin | -0.9231 | -1.5383 | -0.3080 |
| Sydney | -0.0777 | -0.6491 | 0.4937 |
| Dublin | -0.0156 | -0.4035 | 0.3723 |
| Marburg_DFG | -0.2147 | -0.4745 | 0.0450 |
| Marburg_FOR2017 | 2107 | -0.4716 | 0.0502 |
| Ulm/Heidelberg | -0.3289 | -0.9866 | 0.3289 |
| Jena | 0.0237 | -0.2445 | 0.2920 |
| Leipzig | -0.2958 | -0.8563 | 0.2647 |
| Linköping | 0.1166 | -0.1563 | 0.3895 |
| Magdeburg | -0.2535 | -0.7326 | 0.2256 |
| Stanford_MIG | 0.1283 | -0.3664 | 0.6230 |
| Stanford_qMRI | -0.0808 | -0.5904 | 0.4287 |
| Vienna | 0.0391 | -0.2342 | 0.3123 |

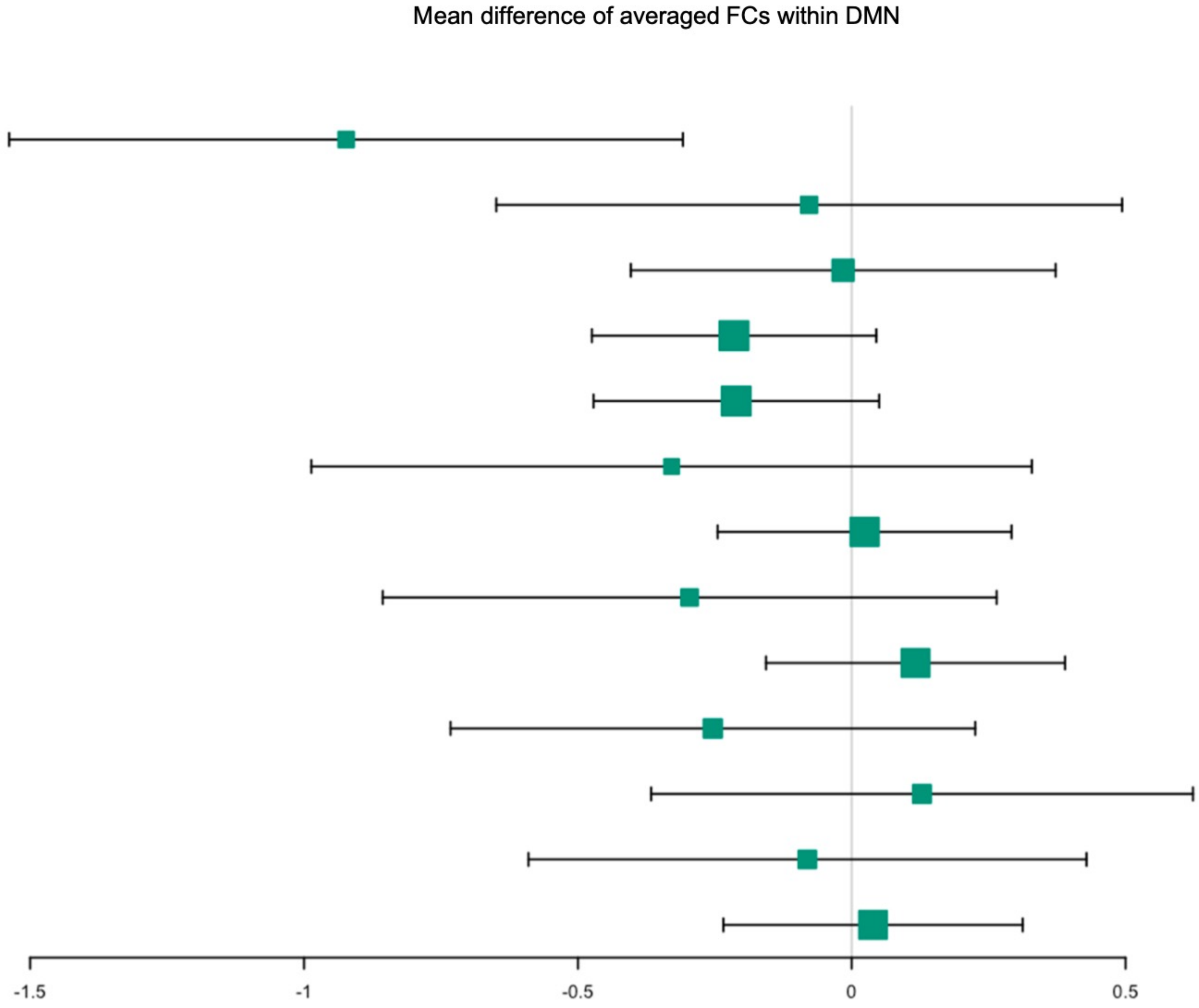

Supplement: Supplementary file 4 — Supplemental Figure 3 [file 41398_2021_1619_MOESM4_ESM.pdf]
