# Supplementary material for: Altered resting-state functional connectome in major depressive disorder: a mega-analysis from the PsyMRI consortium
Source: Transl Psychiatry. 2021 Oct 7;11:511. doi: 10.1038/s41398-021-01619-w (PMC8497531; doi:10.1038/s41398-021-01619-w)

Sites	Adj. mean difference	Confidence interval 2.50%	Confidence interval 97.50%
Berlin	-0.895	-1.636	-0.154
Sydney	-0.372	-0.997	0.254
Dublin	-0.188	-0.513	0.137
Marburg_DFG	-0.276	-0.510	-0.041
Marburg_FOR2017	-0.169	-0.437	0.098
Ulm/Heidelberg	-0.273	-0.954	0.409
Jena	-0.135	-0.421	0.152
Leipzig	-0.080	-0.608	0.447
Linköping	-0.024	-0.218	0.170
Magdeburg	-0.112	-0.552	0.328
Stanford_MIG	0.109	-0.329	0.547
Stanford_qMRI	-0.033	-0.504	0.437
Vienna	-0.019	-0.272	0.234

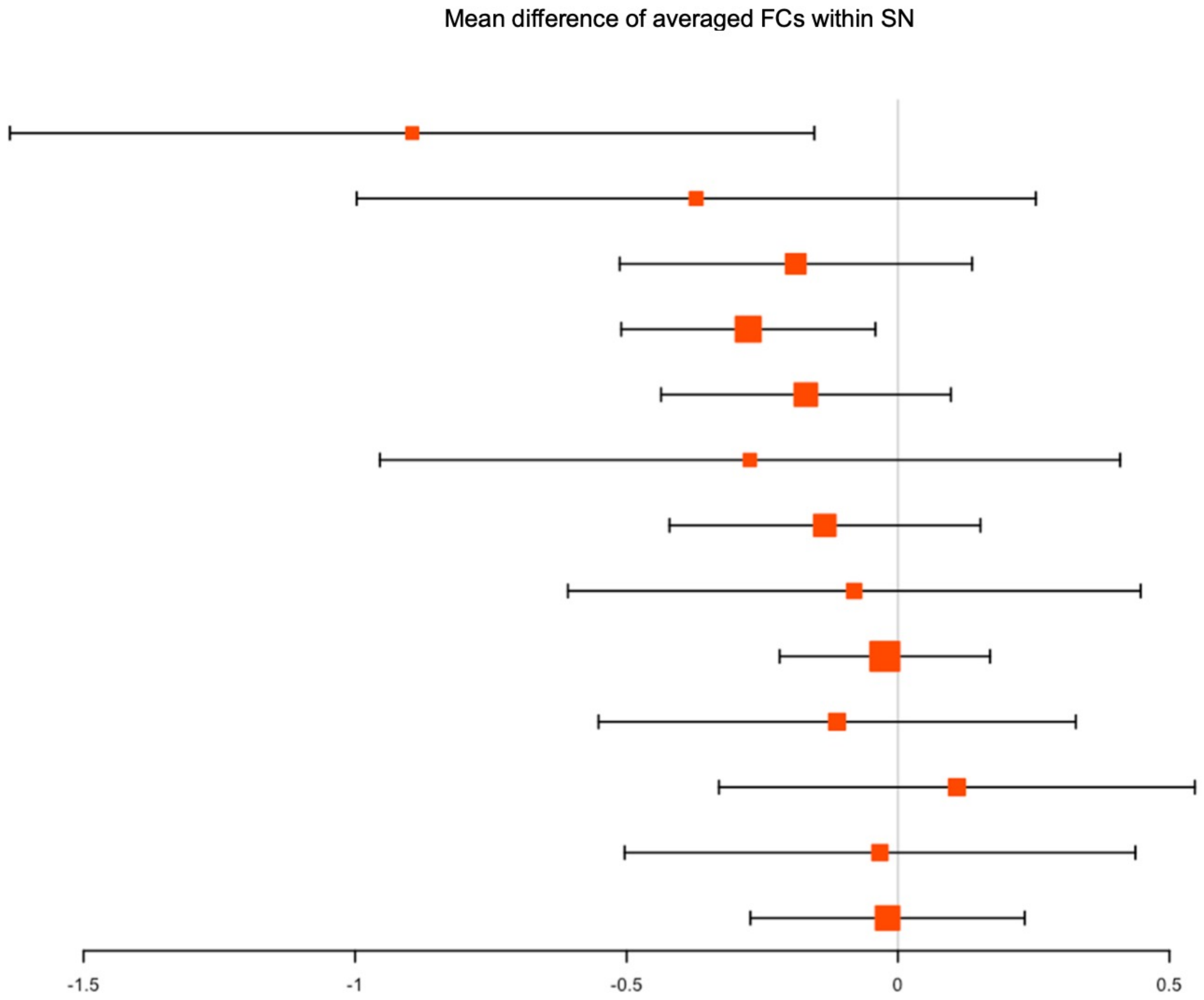

Supplement: Supplementary file 6 — Supplemental Figure 5 [file 41398_2021_1619_MOESM6_ESM.pdf]
